# The real-world safety of Ofatumumab: a pharmacovigilance analysis based on the FDA adverse event reporting system

**DOI:** 10.3389/fimmu.2025.1515730

**Published:** 2025-01-23

**Authors:** Yue Zhou, Yutong Wu, Xiao Zhao, Lingxu Xu, Mingguang Sun, Zhaoyou Meng

**Affiliations:** Department of Neurology, Second Affiliated Hospital of Army Medical University, Chongqing, China

**Keywords:** ofatumumab, FAERS, MS, CLL, pharmacovigilance, adverse events

## Abstract

**Background:**

Ofatumumab is a humanized monoclonal antibody that targets CD20-positive B cells. It was approved by the FDA in 2020 for the treatment of relapsing multiple sclerosis (RMS) in adult patients, and in 2009 for the treatment of Chronic Lymphocytic Leukemia (CLL). With the escalating clinical application of Ofatumumab, comprehending its safety profile within actual healthcare environments is of considerable importance.

**Methods:**

This study compiled a dataset derived from the FAERS database, which included real-world safety data on Ofatumumab from Q4 2009 to Q2 2024. We applied four distinct methodologies, including ROR, PRR, MGPS, and BCPNN, to perform a disproportionality analysis of adverse events (AEs) associated with Ofatumumab. Furthermore, we utilized the Weibull distribution model to estimate the temporal risk pattern of AEs.

**Results:**

The investigation incorporated a total of 24,468 case reports pertaining to AEs associated with Ofatumumab. The commonly observed AEs encompass Fatigue, Headache, Chills, Pyrexia, Pain, Nausea, Nasopharyngitis, Vomiting, Urinary tract infection, and Pneumonia. Additionally, we identified potential AEs not specified on the drug label, such as Asthenia, Hypoesthesia, Dizziness, Malaise, Injection site pain, Paresthesia, and Diarrhea.

**Conclusions:**

This investigation has identified several AEs associated with Ofatumumab and revealed previously unacknowledged potential adverse reaction signals. Healthcare providers can refer to these adverse reaction signals to more comprehensively consider the possible conditions that patients may present with during actual clinical practice.

## 1 Introduction

Ofatumumab is a humanized monoclonal antibody that targets CD20, achieving the effect of clearing B cells from the bloodstream by binding to CD20 antigen. CD20 is a marker specific to B cells, commencing its expression at the Pre-B cell stage and ceasing upon differentiation into plasma cells. It is expressed in over 95% of B-cell malignancies but is not found in hematopoietic stem cells, plasma cells, or other normal tissues (1). Anti-CD20 therapy affects the antigen-presenting function of B cells in multiple sclerosis (MS). Consequently, CD20 is considered an optimal target for the treatment of B-cell lymphomas and autoimmune diseases. As an anti-CD20 antibody, Ofatumumab exhibits binding specificity to non-contiguous segments within the small and large extracellular domains of CD20 antigen, localized at amino acid sequences 74–80 and 145–161 respectively (2), triggering a complement-dependent cytotoxicity (CDC) effect that is more pronounced than antibody-dependent cellular cytotoxicity (ADCC) (3, 4). This results in the formation of a membrane attack complex (MAC) that compromises the cellular membrane integrity of B cells, culminating in cell lysis and apoptosis.

Ofatumumab was approved by the FDA in 2020 for the treatment of relapsing multiple sclerosis (RMS) in adult patients. MS represents an inflammatory demyelinating condition mediated by immune mechanisms within the central nervous system. For a long time, MS was considered to be primarily mediated by autoreactive CD4+ helper T cells. However, recent studies have revealed that B cells play an indispensable and significant role in the development of MS (5). In the context of MS, B lymphocytes participate in the innate immune response, the process of antigen presentation (6), production of regulatory and pro-inflammatory cytokines, chemokines, and autoantibodies (5, 7–9). Research has demonstrated that the maturation of B cells and immune responses are concurrently initiated in both the peripheral and central nervous systems (CNS) (10). In lesions characterized by early and active focal demyelination, CD20+ B cells are predominantly distributed in the perivascular spaces of veins; whereas in progressive MS, a more abundant plasma cell infiltration can be observed in the perivascular spaces (11, 12). Within the meninges, there are B-cell conglomerates, a subset of which exhibit characteristics analogous to lymphoid follicles, and these are associated with regions of subpial demyelination, neuronal depletion, and cortical shrinkage (13). Thus, therapeutic approaches targeting B cells are likely to achieve significant clinical efficacy. The MIRROR study shows that the mean incidence of cumulative new gadolinium-enhancing (GdE) lesions from week 0 to 12 exhibited a 65% decrement that was statistically significant across all Ofatumumab cohorts compared to the placebo group (rate ratio 0.35, 95% [CI] 0.221-0.548, p < 0.001) (14). In the ASCLEPIOS I trial, the adjusted annualized relapse rate was 0.11 for the Ofatumumab group and 0.22 for the teriflunomide group, indicating a substantial difference of -0.11 (95% confidence interval [CI], -0.16 to -0.06; P<0.001). Similarly, in the ASCLEPIOS II trial, the respective rates were 0.10 and 0.25, with a momentous difference of -0.15 (95% CI, -0.20 to -0.09; P<0.001) (15).

Additionally, Ofatumumab was granted approval by the FDA in 2009 for the therapy of Chronic Lymphocytic Leukemia (CLL). CLL is a hematological malignancy characterized by the clonal proliferation of mature B lymphocytes (16). CD20, serving as a specific marker for B-cell lymphomas, plays a significant role in the diagnosis and differentiation of CLL. Nowadays, therapies targeting CD20 remain one of the common treatments for CLL. According to the PROLONG study, in the Ofatumumab group, the median progression-free survival (PFS) was 29.4 months (95% CI 26.2-34.2), compared with 15.2 months in the observation group (11.8-18.8; HR 0.50; 95% CI 0.38–0.66; p<0.0001) (17).

The clinical application of Ofatumumab is relatively broad, with multiple sclerosis (MS) being one of its primary indications. MS impacts over 2.5 million individuals globally (18), and its incidence is noted to be on an upward trend year by year. Consequently, comprehending the safety profile of Ofatumumab in clinical practice is of paramount importance. According to the prescribing information, the most common adverse reactions include upper respiratory tract infections, systemic or local injection-related reactions, headaches, and urinary tract infections. Clinical trials, which encompass specific disease types and stringent inclusion and exclusion criteria, mean that the adverse reactions (AEs) listed in the prescribing information based on these studies may only reflect conditions that may occur in a subset of the population. This study, drawing from the FDA AE reporting system (FAERS) database, provides a comprehensive analysis of the real-world safety profile of Ofatumumab, offering additional evidence for healthcare professionals in their application of Ofatumumab.

## 2 Materials and methods

### 2.1 Data source and processing

**Figure 1:**
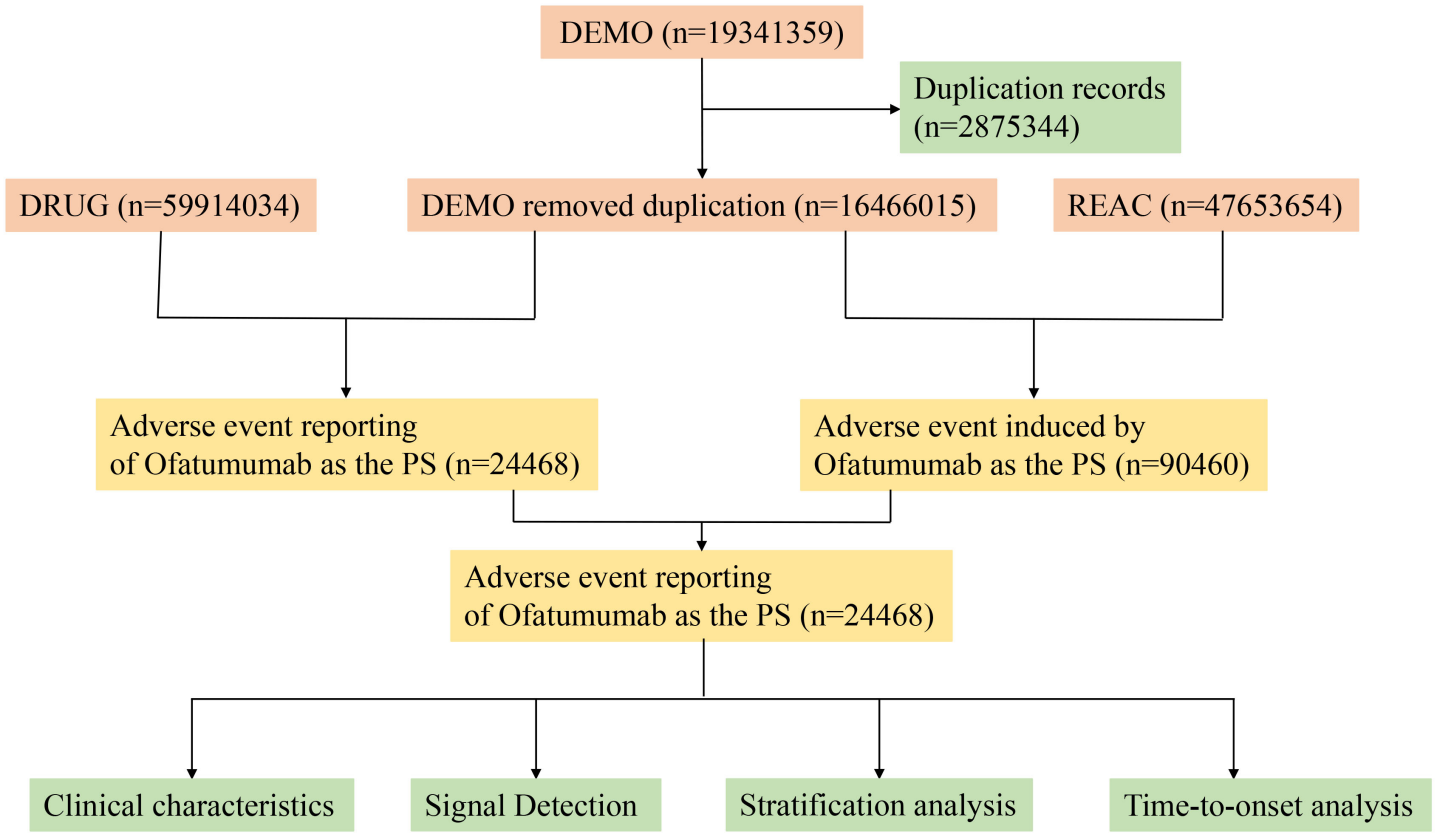
Flow-process diagram of Ofatumumab-related AEs from FAERS database.

This investigation compiled AE reports implicating Ofatumumab as the primary suspect (PS) medication, retrieved from the FAERS database between Q4 2009 and Q2 2024. Data extraction and cleansing were conducted using R version 4.4.1. The database, comprising 19,341,359 reports, was refined by excluding 2,875,344 duplicates, adhering to FDA directives (19). We extracted the PRIMARYID, CASEID, and FDA_DT fields from the DEMO table in the raw data and sorted them. For reports with the same CASEID, we retained the one with the largest FDA_DT value; for reports with both identical CASEID and FDA_DT, we kept the one with the largest PRIMARYID value. AEs were classified according to Preferred Terms (PT) and System Organ Class (SOC) using the Medical Dictionary for Regulatory Activities (MedDRA) version 27.0 (20). **Figure 1** depicts the flow diagram of AEs associated with Ofatumumab from the FAERS database.

### 2.2 Data analysis

In our study, we employed methods including the Reporting Odds Ratio (ROR) (21–23), Proportional Reporting Ratio (PRR) (24), Multi-Item Gamma-Poisson Shrinker (MGPS) (25), and Bayesian Confidence Propagation Neural Network (BCPNN) (26) to assess the significant association between Ofatumumab and AEs. AEs that exceeded the positive response threshold of at least one method were considered potential AEs. **Supplementary Table S1** details the two-by-two contingency matrices, and **Supplementary Table S2** supplements the four primary algorithms used for signal detection. Additionally, the time incidence of AEs was modeled using the Weibull distribution.

## 3 Results

### 3.1 Basic characteristics of AEs and population

The investigation incorporated a total of 24,468 case reports pertaining to AEs associated with Ofatumumab. **Table 1** delineates the baseline data of AE reports associated with Ofatumumab. Females (16,654, 68.1%) constituted a significantly larger portion of the reporters than males (5,830, 23.8%). Among those reporting adverse reactions, adults aged 18-65 years (9,111, 37.2%) formed the largest demographic, followed by individuals over the age of 65 (1475, 6.0%). Consistent with previous statements, the primary indication for Ofatumumab is MS (14,474, 59.2%), with CLL (1,158, 4.7%) as the secondary indication. According to the FAERS database, the most frequently documented serious outcome is hospitalization (2,411, 9.9%), followed by death, life-threatening, and disability. The United States (19,214 78.5%) leads in the number of AE reports, with the United Kingdom (892, 3.6%) in the second position. There is a growing trend among consumers to prioritize health-related concerns, as evidenced by a significant majority of reports (18,914, 77.3%) attributed to consumers. Since the FDA approval of Ofatumumab for the treatment of RMS in 2020, there has been a year-on-year increase in the number of reports related to adverse reactions, with 36.5% recorded in 2023. Should the trend continue, the proportion for 2024 might be higher; however, our statistics only account for the first two quarters.

**Table 1:** Clinical characteristics of Ofatumumab adverse event reports from the FAERS database (Q4 2009 – Q2 2024).

Characteristics	Case numbers	Case proportion (%)
Number of events	24468	
Gender		
Male	5830	23.8
Female	16654	68.1
Miss	1984	8.1
Age		
<18	42	0.2
18-65	9111	37.2
65-85	1422	5.8
>85	53	0.2
Miss	13840	56.6
Top 3 Indications		
Multiple sclerosis	14474	59.2
Chronic lymphocytic leukemia	1158	4.7
B-cell lymphoma	215	0.9
Serious Outcome		
Hospitalization	2411	9.9
Death	537	2.2
Life-Threatening	206	0.8
Disability	106	0.4
Top 5 Reported Countries		
United States	19214	78.5
United Kingdom	892	3.6
Canada	359	1.5
Germany	319	1.3
Japan	317	1.3
Reporter		
Consumer	18914	77.3
Doctor of Medicine	3068	12.5
Healthcare professional	1691	6.9
Pharmacist	236	1.0
Miss	270	1.1
Reporting year		
2009-2020	1930	7.9
2021	3522	14.4
2022	6037	24.7
2023	8930	36.5
2024	4049	16.5

### 3.2 Signal detection related to SOC levels

**Figure 2:**
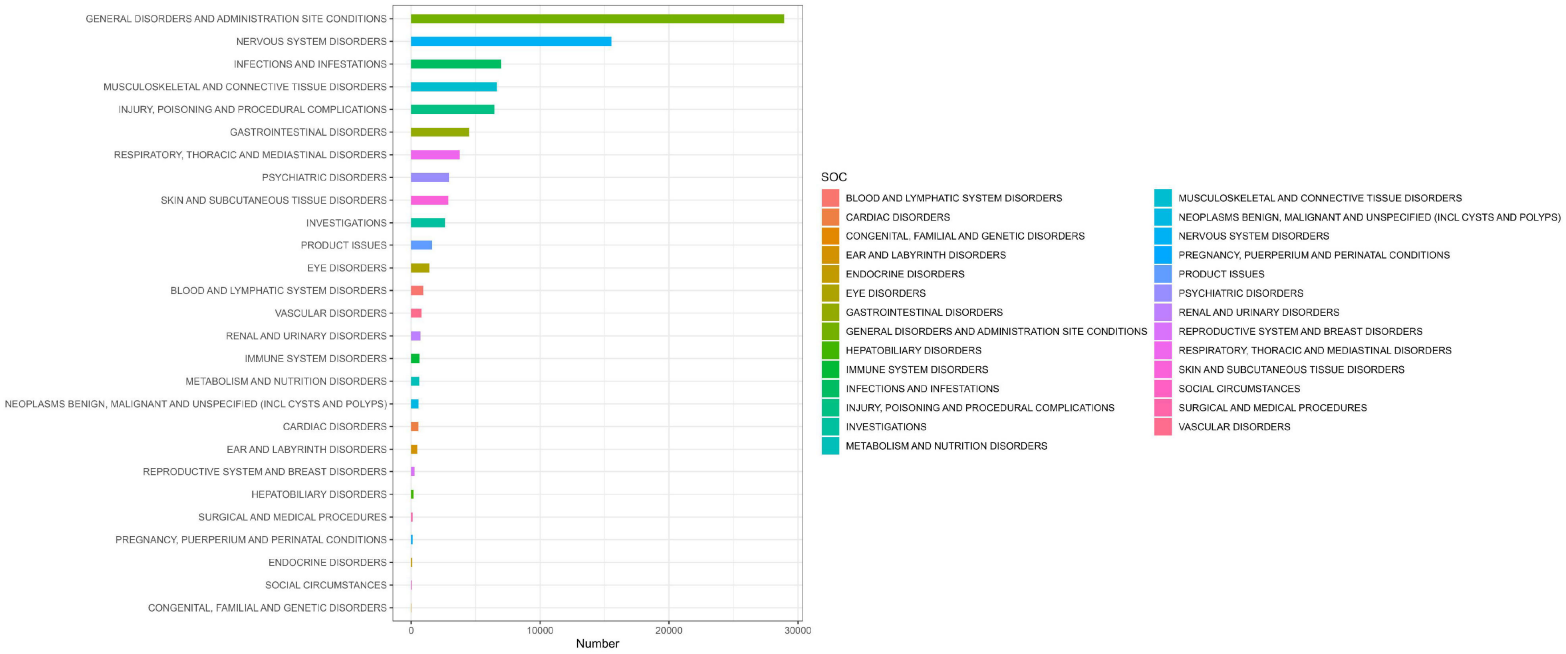
AEs related to the system organ class (SOC) level.

**Figure 3:**
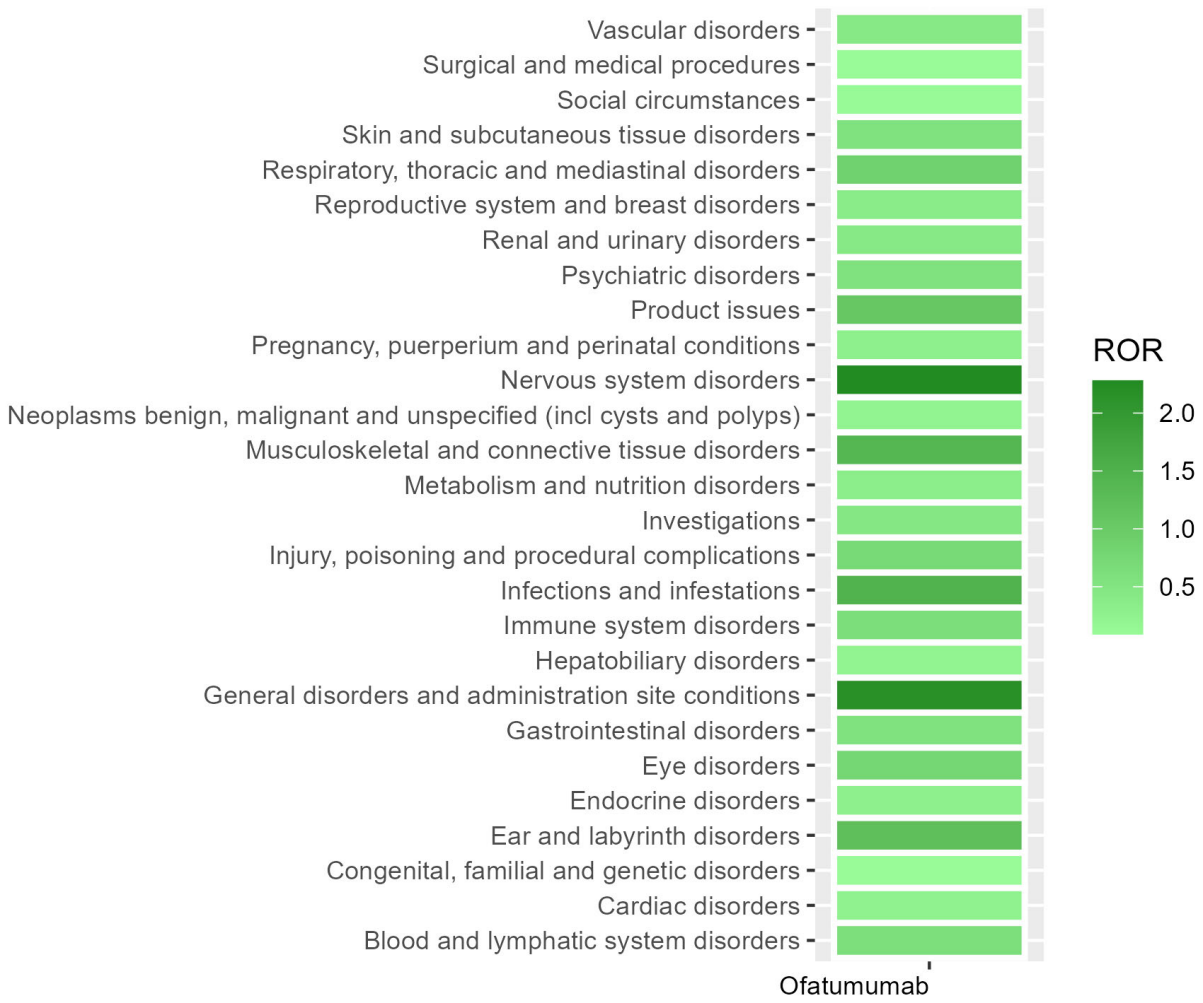
Heatmap of SOC level.

**Table 2** presents the AEs associated with Ofatumumab, covering all 27 SOCs, while **Figures 2**-**3** illustrates the signal strength of Ofatumumab within the SOC levels in the FAERS database. The most commonly reported category was General disorders and administration site conditions [n = 28,934, ROR (95%CI) = 2.18 (2.15-2.21)], whereas the category with the strongest signal intensity was Nervous system disorders [n = 15,525, ROR (95%CI) = 2.28 (2.24-2.32)]. Several additional SOCs have demonstrated robust signal detection, including Infections and infestations [n = 6,993, ROR (95%CI) = 1.48 (1.45-1.52)], Musculoskeletal and connective tissue disorders [n = 6,636, ROR (95%CI) = 1.4 (1.36-1.43)], Ear and labyrinth disorders [n = 473, ROR (95%CI) = 1.21 (1.11-1.33)], Product issues [n = 1,618, ROR (95%CI) = 1.06 (1.01-1.11)]. The lack of statistical significance is due to the lower bounds of the ROR (95% CI) falling below 1, such as Congenital, familial and genetic disorders, Surgical and medical procedures, Social circumstances, etc.

**Table 2:** Signal strength of Ofatumumab AEs across System Organ Classes (SOC) in the FAERS database.

System Organ Class (SOC)	Case numbers	ROR(95%CI)	PRR(χ^2^)	EBGM(EBGM05)	IC(IC025)
General disorders and administration site conditions*	28934	2.18 (2.15 - 2.21)	1.8 (12483.24)	1.8 (1.78)	0.85 (-0.82)
Nervous system disorders*	15525	2.28 (2.24 - 2.32)	2.06 (9203.38)	2.06 (2.03)	1.04 (-0.63)
Infections and infestations*	6993	1.48 (1.45 - 1.52)	1.44 (1008.48)	1.44 (1.41)	0.53 (-1.14)
Musculoskeletal and connective tissue disorders*	6636	1.4 (1.36 - 1.43)	1.37 (699.43)	1.37 (1.34)	0.45 (-1.21)
Injury, poisoning and procedural complications	6466	0.71 (0.69 - 0.73)	0.73 (723.19)	0.73 (0.71)	-0.46 (-2.12)
Gastrointestinal disorders	4492	0.56 (0.54 - 0.58)	0.58 (1467.34)	0.58 (0.57)	-0.78 (-2.44)
Respiratory, thoracic and mediastinal disorders	3759	0.87 (0.84 - 0.9)	0.88 (70.08)	0.88 (0.85)	-0.19 (-1.86)
Psychiatric disorders	2921	0.56 (0.54 - 0.58)	0.58 (965.65)	0.58 (0.56)	-0.8 (-2.46)
Skin and subcutaneous tissue disorders	2868	0.57 (0.54 - 0.59)	0.58 (926.63)	0.58 (0.56)	-0.79 (-2.45)
Investigations	2614	0.47 (0.46 - 0.49)	0.49 (1474.66)	0.49 (0.47)	-1.03 (-2.69)
Product issues*	1618	1.06 (1.01 - 1.11)	1.06 (4.96)	1.06 (1.01)	0.08 (-1.59)
Eye disorders	1402	0.77 (0.73 - 0.81)	0.77 (95.24)	0.77 (0.74)	-0.37 (-2.04)
Blood and lymphatic system disorders	945	0.62 (0.58 - 0.66)	0.63 (212.17)	0.63 (0.6)	-0.67 (-2.34)
Vascular disorders	809	0.42 (0.39 - 0.45)	0.43 (638.33)	0.43 (0.4)	-1.23 (-2.9)
Renal and urinary disorders	716	0.42 (0.39 - 0.45)	0.42 (573.14)	0.42 (0.4)	-1.24 (-2.9)
Immune system disorders	650	0.63 (0.58 - 0.68)	0.63 (140.74)	0.63 (0.59)	-0.66 (-2.33)
Metabolism and nutrition disorders	630	0.33 (0.3 - 0.35)	0.33 (874.55)	0.33 (0.31)	-1.6 (-3.26)
Neoplasms benign, malignant and unspecified (incl cysts and polyps)	575	0.22 (0.21 - 0.24)	0.23 (1532.61)	0.23 (0.21)	-2.12 (-3.79)
Cardiac disorders	562	0.25 (0.23 - 0.27)	0.25 (1269.98)	0.25 (0.24)	-1.98 (-3.65)
Ear and labyrinth disorders*	473	1.21 (1.11 - 1.33)	1.21 (17.44)	1.21 (1.12)	0.28 (-1.39)
Reproductive system and breast disorders	275	0.37 (0.33 - 0.42)	0.37 (295.8)	0.37 (0.34)	-1.43 (-3.1)
Hepatobiliary disorders	186	0.23 (0.2 - 0.27)	0.23 (470.05)	0.23 (0.21)	-2.09 (-3.76)
Surgical and medical procedures	112	0.09 (0.07 - 0.11)	0.09 (1041.04)	0.09 (0.08)	-3.47 (-5.13)
Pregnancy, puerperium and perinatal conditions	112	0.3 (0.25 - 0.36)	0.3 (183.72)	0.3 (0.26)	-1.74 (-3.4)
Endocrine disorders	69	0.3 (0.24 - 0.38)	0.3 (113.79)	0.3 (0.25)	-1.74 (-3.41)
Social circumstances	38	0.1 (0.07 - 0.14)	0.1 (313.38)	0.1 (0.08)	-3.34 (-5)
Congenital, familial and genetic disorders	25	0.09 (0.06 - 0.14)	0.09 (224.29)	0.09 (0.07)	-3.44 (-5.1)

Asterisks (*) indicate statistically significant signals in algorithm; ROR, reporting odds ratio; PRR, proportional reporting ratio; EBGM, empirical Bayesian geometric mean; EBGM05, the lower limit of the 95% CI of EBGM; IC, information component; IC025, the lower limit of the 95% CI of the IC; CI, confidence interval; AEs, adverse events.

### 3.3 Signal detection related to PT levels

**Figure 4:**
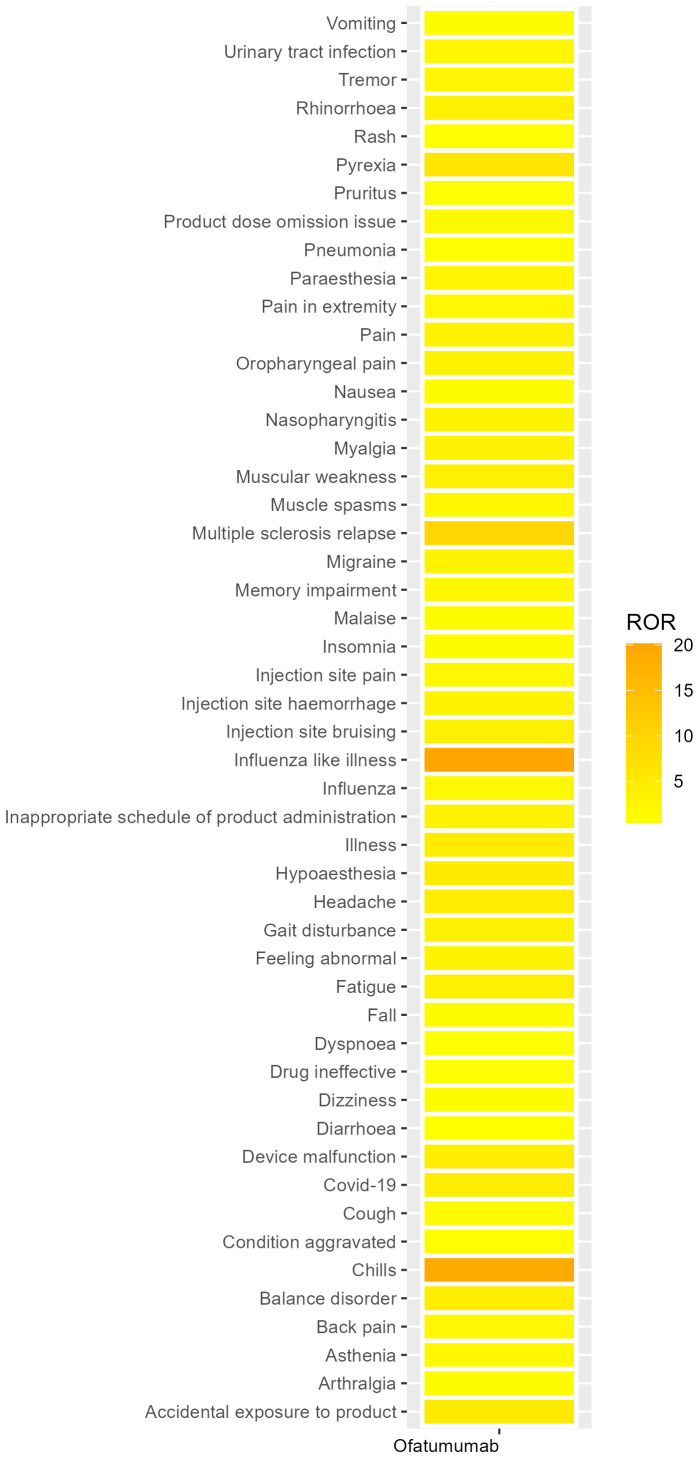
Heatmap of PT level.

We systematically categorized all AEs related to Ofatumumab by their frequency, with the results methodically detailed in **Table 3**. **Figure 4** presents the intensity of Ofatumumab signals across various PT categories as documented in the FAERS database. Additionally, all AEs fulfilling the positivity signal criteria have been meticulously recorded in **Supplementary Table S3**. The top 10 PTs were comprised of Fatigue, Headache, Chills, Pyrexia, Pain, Influenza like illness, Nausea, Covid-19, Asthenia, Hypoaesthesia. Some PTs are enumerated in the drug label, including Fatigue, Headache, Chills, Pyrexia, Pain, Nausea, Nasopharyngitis, Vomiting, Urinary tract infection, Pneumonia, etc. There also exist potential adverse reactions that are not mentioned within the drug’s prescribing information, such as Asthenia, Hypoaesthesia, Dizziness, Malaise, Injection site pain, Paraesthesia, Diarrhoea, etc.

**Table 3:** Top 50 frequency of adverse events at the PT level for Ofatumumab.

PT	Case numbers	ROR(95%CI)	PRR(χ^2^)	EBGM(EBGM05)	IC(IC025)
Fatigue*	4566	3.99 (3.87 - 4.11)	w3.84 (9642.74)	3.82 (3.72)	1.93 (0.27)
Headache*	3973	4.35 (4.22 - 4.49)	4.21 (9733.13)	4.18 (4.07)	2.06 (0.4)
Chills*	3121	19.19 (18.5 - 19.9)	18.56 (50182.14)	17.96 (17.42)	4.17 (2.5)
Pyrexia*	2992	6.1 (5.88 - 6.33)	5.93 (12200.13)	5.88 (5.7)	2.56 (0.89)
Pain*	2963	3.09 (2.98 - 3.21)	3.03 (4039.22)	3.01 (2.92)	1.59 (-0.07)
Influenza like illness*	2416	20.15 (19.33 - 20.99)	19.63 (41248.7)	18.96 (18.32)	4.25 (2.58)
Nausea*	1472	1.28 (1.21 - 1.34)	1.27 (86.55)	1.27 (1.22)	0.35 (-1.32)
Covid-19*	1244	4.4 (4.16 - 4.65)	4.35 (3197.74)	4.33 (4.13)	2.11 (0.45)
Asthenia*	1161	2.11 (1.99 - 2.24)	2.1 (666.51)	2.09 (1.99)	1.06 (-0.6)
Hypoaesthesia*	1069	4.81 (4.52 - 5.11)	4.76 (3156.96)	4.73 (4.5)	2.24 (0.58)
Gait disturbance*	1064	3.54 (3.33 - 3.76)	3.51 (1900.29)	3.49 (3.32)	1.8 (0.14)
Multiple sclerosis relapse*	1064	9.34 (8.78 - 9.92)	9.24 (7691.69)	9.1 (8.64)	3.19 (1.52)
Feeling abnormal*	1043	2.83 (2.66 - 3.01)	2.81 (1213.13)	2.8 (2.66)	1.48 (-0.18)
Dizziness*	997	1.37 (1.29 - 1.46)	1.37 (98.74)	1.37 (1.3)	0.45 (-1.22)
Malaise*	985	1.42 (1.33 - 1.51)	1.41 (119.95)	1.41 (1.34)	0.5 (-1.17)
Drug ineffective	913	0.44 (0.41 - 0.47)	0.45 (645.67)	0.45 (0.42)	-1.17 (-2.83)
Pain in extremity*	896	1.96 (1.83 - 2.09)	1.95 (413.37)	1.94 (1.84)	0.96 (-0.71)
Nasopharyngitis*	880	3.11 (2.91 - 3.33)	3.09 (1242.73)	3.08 (2.91)	1.62 (-0.04)
Injection site pain*	845	1.93 (1.81 - 2.07)	1.93 (376.61)	1.92 (1.82)	0.94 (-0.72)
Arthralgia*	759	1.21 (1.13 - 1.3)	1.21 (27.38)	1.21 (1.14)	0.27 (-1.39)
Myalgia*	741	3.03 (2.81 - 3.25)	3.01 (991.5)	3 (2.82)	1.58 (-0.08)
Inappropriate schedule of product administration*	735	3.02 (2.81 - 3.25)	3.01 (981.87)	3 (2.82)	1.58 (-0.08)
Back pain*	723	2.05 (1.91 - 2.21)	2.04 (385.75)	2.04 (1.92)	1.03 (-0.64)
Accidental exposure to product*	706	5.18 (4.81 - 5.58)	5.14 (2337.54)	5.1 (4.8)	2.35 (0.69)
Vomiting	698	1.04 (0.96 - 1.12)	1.04 (0.85)	1.04 (0.97)	0.05 (-1.62)
Cough*	686	1.63 (1.51 - 1.76)	1.63 (165.57)	1.62 (1.52)	0.7 (-0.97)
Fall*	685	1.37 (1.27 - 1.47)	1.36 (66.87)	1.36 (1.28)	0.45 (-1.22)
Muscle spasms*	640	2.29 (2.12 - 2.48)	2.28 (460.56)	2.28 (2.13)	1.19 (-0.48)
Muscular weakness*	630	3.7 (3.42 - 4.01)	3.68 (1225.7)	3.67 (3.43)	1.87 (0.21)
Product dose omission issue*	628	1.79 (1.65 - 1.93)	1.78 (214.86)	1.78 (1.66)	0.83 (-0.84)
Paraesthesia*	621	2.64 (2.44 - 2.86)	2.63 (625.98)	2.62 (2.45)	1.39 (-0.28)
Urinary tract infection*	619	2.41 (2.22 - 2.6)	2.4 (502.67)	2.39 (2.24)	1.26 (-0.41)
Tremor*	600	2.49 (2.3 - 2.7)	2.48 (529.59)	2.47 (2.31)	1.31 (-0.36)
Illness*	563	4.51 (4.15 - 4.9)	4.48 (1512.96)	4.45 (4.15)	2.16 (0.49)
Balance disorder*	554	4.18 (3.84 - 4.54)	4.16 (1321.04)	4.13 (3.85)	2.05 (0.38)
Memory impairment*	503	2.33 (2.14 - 2.55)	2.33 (379.47)	2.32 (2.16)	1.21 (-0.45)
Oropharyngeal pain*	485	3.3 (3.01 - 3.6)	3.28 (766.48)	3.27 (3.03)	1.71 (0.04)
Injection site bruising*	457	3.95 (3.6 - 4.33)	3.94 (995.54)	3.92 (3.63)	1.97 (0.3)
Insomnia*	444	1.12 (1.02 - 1.23)	1.12 (5.51)	1.12 (1.03)	0.16 (-1.51)
Diarrhoea	441	0.45 (0.41 - 0.5)	0.46 (288.86)	0.46 (0.42)	-1.13 (-2.8)
Migraine*	434	3.1 (2.82 - 3.41)	3.09 (610.09)	3.08 (2.84)	1.62 (-0.04)
Dyspnoea	432	0.51 (0.46 - 0.56)	0.51 (203.51)	0.51 (0.47)	-0.97 (-2.63)
Rash	427	0.67 (0.61 - 0.74)	0.67 (69.44)	0.67 (0.62)	-0.58 (-2.24)
Pruritus	422	0.79 (0.72 - 0.87)	0.8 (22.22)	0.8 (0.73)	-0.33 (-1.99)
Device malfunction*	414	4.19 (3.8 - 4.61)	4.17 (992.23)	4.15 (3.83)	2.05 (0.39)
Pneumonia	409	0.85 (0.77 - 0.93)	0.85 (11.4)	0.85 (0.78)	-0.24 (-1.91)
Condition aggravated	400	0.94 (0.85 - 1.03)	0.94 (1.77)	0.94 (0.86)	-0.1 (-1.76)
Rhinorrhoea*	382	3.92 (3.55 - 4.34)	3.91 (822.37)	3.89 (3.57)	1.96 (0.29)
Injection site haemorrhage*	356	3.09 (2.78 - 3.43)	3.08 (497.45)	3.07 (2.81)	1.62 (-0.05)
Influenza*	347	2.09 (1.88 - 2.32)	2.08 (195.26)	2.08 (1.9)	1.06 (-0.61)

Asterisks (*) indicate statistically significant signals in algorithm; ROR, reporting odds ratio; PRR, proportional reporting ratio; EBGM, empirical Bayesian geometric mean; EBGM05, the lower limit of the 95% CI of EBGM; IC, information component; IC025, the lower limit of the 95% CI of the IC; CI, confidence interval; PT, preferred term.

### 3.4 Information from subgroup

**Supplementary Tables S4**-**11** provide a detailed explanation of the subgroup analyses related to Ofatumumab. In the gender subgroup, both males and females most frequently reported Fatigue; additionally, males were more likely to experience Chills, while females were more prone to Headache. Within the age subgroups, individuals under 18 years old exhibited a higher incidence of Nephrotic syndrome, adults aged 18 to 65 were most susceptible to Fatigue, and those over 65 years old frequently experienced Pyrexia. Consumers are often acutely aware of their own Fatigue, whereas medical personnel are more inclined to report Pyrexia.

### 3.5 Sensitivity analysis

Based on the baseline data in the FAERS database, the primary indication for Ofatumumab is MS. Consequently, we have excluded certain concomitant medications that may be used in the treatment of MS, including glucocorticoids, teriflunomide, fingolimod hydrochloride, siponimod, ozanimod, dimethyl fumarate, glatiramer acetate, and mitoxantrone. Following the exclusion of cases necessitating supplementary concurrent drug therapy, a total of 23,368 case reports were detected, encompassing 86,065 AEs. The primary adverse reactions reported include Fatigue, Headache, Chills, Pain, and Pyrexia (Details can be found in **Supplementary Table 12**).

### 3.6 Time-to-onset and weibull distribution analysis of AEs based on ofatumumab

**Figure 5:**
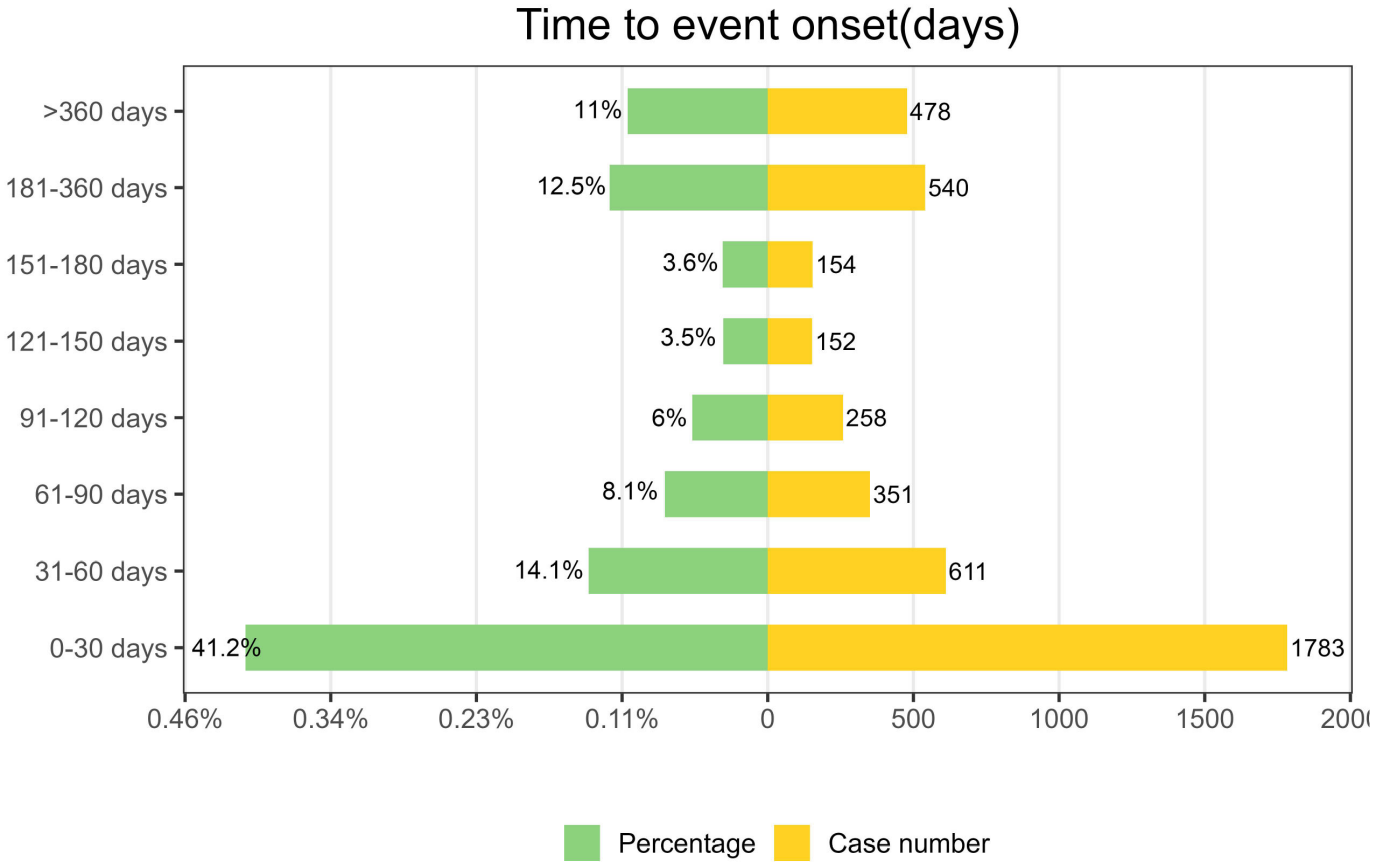
Time-to-onset of AEs.

As depicted in **Figure 5**, AEs associated with Ofatumumab predominantly occur within the first 30 days of therapeutic intervention. Additionally, the Weibull distribution analysis revealed a pattern of early failures, with the specific parameters detailed in **Table 4**.

**Table 4:** Time to onset of Ofatumumab-associated adverse events and Weibull distribution analysis.

Drug	TTO(days)		Weibull distribution		
	Case reports	Median(d) (IQR)	Scale parameter: α(95%CI)	Shape parameter: β(95%CI)	Type
Ofatumumab	4327	49 (12-169)	95.64 (90.84-100.44)	0.63 (0.61-0.64)	Early failure

TTO, time to onset; CI, contrialfidence interval; IQR, interquartile range.

## 4 Discussion

This investigation analyzed the AEs related to Ofatumumab, as recorded in the FAERS database from its FDA approval and market introduction in Q4 2009. The findings substantiated various adverse reactions previously noted in the drug’s prescribing information, such as Fatigue, Headache, Chills, Pyrexia, Pain, Nausea, Nasopharyngitis, Vomiting, Urinary tract infection, and Pneumonia. Furthermore, the study identified additional adverse reactions not yet incorporated into the prescribing information, including those within the categories of General disorders and administration site conditions, Infections and infestations, Nervous system disorders, and Gastrointestinal disorders.

Studies indicate that the onset of MS typically occurs between the ages of 20 and 40, making it the most common cause of neurological disability among young adults, with a prevalence in females approximately twice that of males (27, 28). Consequently, in our baseline data, the number of adverse reactions reported by females significantly exceeds that of males. Additionally, the proportion of AE reports among individuals aged 18-65 is the highest compared to other age groups. The number of adverse reaction cases reported in the United States greatly surpasses those from other countries or regions, likely due to the FAERS being a domestic reporting system for adverse reactions in the U.S. Furthermore, research has shown that the prevalence of MS among adults in the U.S. is highest in whites (374.8 per 100,000; 95% CI 373.8-375.8) (29), which may also account for the high proportion of 78.5% reported in the U.S. among the countries surveyed. With the widespread dissemination of health education, people today place greater emphasis on health-related issues than in the past, leading to consumers reporting the highest number of adverse reaction signals. Other possible reasons include the fact that Ofatumumab, used for treating MS, is packaged as an auto-injector pen for subcutaneous injection, allowing patients to administer the medication without frequent visits to medical facilities.

In the category of general disorders and administration site conditions, fatigue, chills, pyrexia, and pain are listed in the drug’s prescribing information, whereas asthenia, malaise, and injection site pain have not yet been included in the drug label. Ofatumumab is capable of eliciting a robust CDC response, which is considered a critical factor in the initiation of infusion-related reactions (30), subsequently resulting in undesirable outcomes such as pyrexia. The incidence of systemic and local reactions associated with injections is generally highest during the initial injection and decreases with subsequent injections, with the severity typically being mild to moderate. In the MIRROR trial, the most common AEs from week 0 to 12 in the Ofatumumab group were injection-related reactions (IRRs), with a prevalence of 41% to 66%, primarily associated with the first dose of Ofatumumab (29%-50%) (14). Although these adverse reactions do not lead to severe outcomes, they frequently occur during the long-term treatment of patients, affecting their compliance and trust in healthcare professionals. In the real-world clinical setting, we can enhance communication with patients and promptly address their symptoms to achieve better clinical outcomes.

In our study, Infections and infestations are also significant among the adverse events caused by Ofatumumab. In the ASCLEPIOS I trial, Infections and infestations were reported in 229 patients (49.2%) who received Ofatumumab. As well as the ASCLEPIOS II research, recipients reported infections and infestations in 259 patients, accounting for 53.8% of the total. In the two trials, infections reported by at least 10% of patients in both groups included nasopharyngitis (18.0%), upper respiratory tract infections (10.3%), and urinary tract infections (10.3%) (15). In the PROLONG study, the incidence of pneumonia was observed in 11.0% of the patient cohort who was treated with Ofatumumab (17). Patients undergoing anti-CD20 therapy may have underlying hypogammaglobulinemia. Persistent hypogammaglobulinemia over the long term is considered to increase the risk of infection (31). As of now, no instances have been documented where progressive multifocal leukoencephalopathy (PML) has developed in MS patients receiving Ofatumumab therapy. However, in a clinical trial investigating the combination therapy of idelalisib with Ofatumumab for previously treated CLL, there were cases of PML infections that led to mortality (32). Treatment-induced immunosuppression may lead to the reactivation of latent JC virus (JCV), thereby causing progressive multifocal leukoencephalopathy (33). Accordingly, while administering Ofatumumab therapy, there should be a proactive approach to infection prevention, and any identified infections should be promptly addressed to prevent or decelerate disease progression.

Nervous system disorders also constitute a frequently reported category of adverse reactions. During the treatment of MS with Ofatumumab, the adverse reaction of headache is commonly observed which has been documented in the prescribing information. In the APLIOS trial, the probability of patients experiencing headache can reach 12.7% (34). In some clinical trials, the occurrence of headaches is often considered to be associated with systemic injection-related reactions (15, 34). Interestingly, a meta-analysis indicates that B-cell targeted therapy shows a trend towards an increased risk of headaches, yet this trend does not reach statistical significance (35). Additionally, studies have indicated that midbrain/periaqueductal gray (PAG) MS lesions may also be correlated with an increased incidence of headaches (36, 37). Based on previous research, headaches may be one of the adverse effects of ofatumumab treatment for MS, and they may also be a clinical manifestation of the disease progression itself. The data currently available in the FAERS database do not allow for a clear distinction between these two possibilities. According to the FAERS database, we also have observed adverse reactions of hypoesthesia, dizziness, and paresthesia in some patients that are not documented in the drug’s prescribing information, and these reactions are often disregarded in clinical trials due to their mild manifestation. There are numerous potential causes for adverse reactions associated with neurological disorders. During the application of medication, we should make careful assessments and determine the subsequent treatment plan for patients based on the actual circumstances.

In numerous clinical studies on Ofatumumab treatment for CLL, neutropenia is observed as a common adverse reaction not mentioned in the drug’s prescribing information, nor does it appear among the top 50 most frequent adverse reaction signals in the FAERS database at the PT level. The likely reason is that Ofatumumab has been frequently used for the treatment of MS in recent years, while its application in CLL treatment is relatively less common. The depletion of CD20-positive B cells can lead to varying degrees of neutropenia. However, the limited reduction of neutrophils induced by ofatumumab in MS may be related to the route of administration and the dosage used (38). Patients with MS often experience adverse reactions such as infections and injection-related reactions, whereas neutropenia is a type of hematological adverse reaction. This also suggests that medical personnel should conduct a comprehensive assessment of patients when prescribing medications in clinical practice, aiming to minimize the occurrence of various AEs.

By conducting a sensitivity analysis, we have mitigated the influence of glucocorticoids, teriflunomide, fingolimod hydrochloride, siponimod, ozanimod, dimethyl fumarate, glatiramer acetate, and mitoxantrone, all of which are typically administered alongside Ofatumumab in MS therapy and are extensively utilized in standard clinical settings. The adverse reactions specifically linked to Ofatumumab monotherapy encompass fatigue, headache, chills, pain, pyrexia, and nausea. Continuous surveillance of adverse reaction signals contributes to a more thorough evaluation of patient conditions and supports the determination of subsequent therapeutic approaches.

In this study, a temporal analysis of AEs was conducted, employing the Weibull distribution model to project the likely timing of these events. The preponderance of adverse reactions was observed to emerge within the initial month of therapeutic intervention. This highlights the critical nature of early identification and underscores the necessity for vigilant patient monitoring for symptomatic alterations in the early stages of treatment within our standard medical practice.

Our study has certain limitations. Firstly, the FAERS database primarily relies on voluntary reporting by consumers and healthcare professionals, which is susceptible to underreporting, duplicate reporting, and inaccurate reporting, thus introducing bias, such as selection bias. Secondly, the FAERS database does not categorize the severity of adverse reactions, nor does it differentiate between adverse reactions due to other diseases or other treatments, serving only as an indicator. Some symptoms inherent to certain diseases may also be reported as AEs. Clinical staff still need to make judgments based on actual conditions during the diagnosis and treatment process. Thirdly, the FAERS database only reflects the effects after a single stage of treatment, lacking long-term follow-up information, making it difficult to assess the long-term safety of drugs in the real world. Additionally, the FAERS database mainly collects adverse reaction reports from within the United States, which presents regional limitations and makes it difficult to widely collect data related to diseases with low incidence rates in the US region.

## 5 Conclusion

This study leveraged the FDA Adverse Event Reporting System (FAERS) database to perform a pharmacovigilance analysis of Ofatumumab’s safety in real-world settings. The commonly observed AEs encompassed Fatigue, Headache, Chills, Pyrexia, Pain, Nausea, Nasopharyngitis, Vomiting, Urinary tract infection, and Pneumonia. Additionally, we identified potential AEs not specified on the drug label, such as Asthenia, Hypoesthesia, Dizziness, Malaise, Injection site pain, Paresthesia, and Diarrhea. Healthcare providers can refer to these adverse reaction signals to more comprehensively consider the possible conditions that patients may present with during actual clinical practice.

## Data Availability

The original contributions presented in the study are included in the article/**Supplementary Material**. Further inquiries can be directed to the corresponding author.
