# Supplementary material for: The real-world safety of Ofatumumab: a pharmacovigilance analysis based on the FDA adverse event reporting system
Source: Front Immunol. 2025 Jan 23;16:1515730. doi: 10.3389/fimmu.2025.1515730 (PMC11798770; doi:10.3389/fimmu.2025.1515730)
Supplement: Supplementary file 1 [file Table1.docx]

Supplementary Material

# Supplementary Tables

**Supplementary Figure 1** Two-by-two contingency table for disproportionality analyses.

|  | Target AEs | Other AEs | Total |
| --- | --- | --- | --- |
| Target drugs | a | b | a+b |
| Other drugs | c | d | c+d |
| Total | a+c | b+d | a+b+c+d |

## Abbreviation: AEs, adverse events; a, number of reports containing both the target drug and target adverse drug reaction; b, number of reports containing other adverse drug reaction of the target drug; c, number of reports containing the target adverse drug reaction of other drugs; d, number of reports containing other drugs and other adverse drug reactions.

**Supplementary Figure 2** Four major algorithms used for signal detection.

| Algorithms | Equation | Criteria |
| --- | --- | --- |
| ROR | ROR=ad/b/c | lower limit of 95% CI>1, N≥3 |
|  | 95%CI=e^ln(ROR)±1.96(1/a+1/b+1/c+1/d)^0.5^ |  |
| PRR | PRR=a(c+d)/c/(a+b) | PRR≥2, χ^2^≥4, N≥3 |
|  | χ^2^=[(ad-bc)^2](a+b+c+d)/[(a+b)(c+d)(a+c)(b+d)] |  |
| BCPNN | IC=log_2_a(a+b+c+d)(a+c)(a+b) | IC025>0 |
|  | 95%CI= E(IC) ± 2V(IC)^0.5 |  |
| MGPS | EBGM=a(a+b+c+d)/(a+c)/(a+b) | EBGM05>2 |
|  | 95%CI=e^ln(EBGM)±1.96(1/a+1/b+1/c+1/d)^0.5^ |  |

Abbreviation: a, number of reports containing both the target drug and target adverse drug reaction; b, number of reports containing other adverse drug reaction of the target drug; c, number of reports containing the target adverse drug reaction of other drugs; d, number of reports containing other drugs and other adverse drug reactions. 95%CI, 95% confidence interval; N, the number of reports; χ2, chi-squared; IC, information component; IC025, the lower limit of 95% CI of the IC; E(IC), the IC expectations; V(IC), the variance of IC; EBGM, empirical Bayesian geometric mean; EBGM05, the lower limit of 95% CI of EBGM.

**Supplementary Table 3** All adverse events meeting the positive signal threshold at the PT level from FAERS data**.**

| PT | Case numbers | ROR(95%CI) | PRR(χ^2^) | EBGM(EBGM05) | IC(IC025) |
| --- | --- | --- | --- | --- | --- |
| Fatigue | 4566 | 3.99 ( 3.87 - 4.11 ) | 3.84 ( 9642.74 ) | 3.82 ( 3.72 ) | 1.93 ( 0.27 ) |
| Headache | 3973 | 4.35 ( 4.22 - 4.49 ) | 4.21 ( 9733.13 ) | 4.18 ( 4.07 ) | 2.06 ( 0.4 ) |
| Chills | 3121 | 19.19 ( 18.5 - 19.9 ) | 18.56 ( 50182.14 ) | 17.96 ( 17.42 ) | 4.17 ( 2.5 ) |
| Pyrexia | 2992 | 6.1 ( 5.88 - 6.33 ) | 5.93 ( 12200.13 ) | 5.88 ( 5.7 ) | 2.56 ( 0.89 ) |
| Influenza like illness | 2416 | 20.15 ( 19.33 - 20.99 ) | 19.63 ( 41248.7 ) | 18.96 ( 18.32 ) | 4.25 ( 2.58 ) |
| Covid-19 | 1244 | 4.4 ( 4.16 - 4.65 ) | 4.35 ( 3197.74 ) | 4.33 ( 4.13 ) | 2.11 ( 0.45 ) |
| Hypoaesthesia | 1069 | 4.81 ( 4.52 - 5.11 ) | 4.76 ( 3156.96 ) | 4.73 ( 4.5 ) | 2.24 ( 0.58 ) |
| Multiple sclerosis relapse | 1064 | 9.34 ( 8.78 - 9.92 ) | 9.24 ( 7691.69 ) | 9.1 ( 8.64 ) | 3.19 ( 1.52 ) |
| Gait disturbance | 1064 | 3.54 ( 3.33 - 3.76 ) | 3.51 ( 1900.29 ) | 3.49 ( 3.32 ) | 1.8 ( 0.14 ) |
| Accidental exposure to product | 706 | 5.18 ( 4.81 - 5.58 ) | 5.14 ( 2337.54 ) | 5.1 ( 4.8 ) | 2.35 ( 0.69 ) |
| Muscular weakness | 630 | 3.7 ( 3.42 - 4.01 ) | 3.68 ( 1225.7 ) | 3.67 ( 3.43 ) | 1.87 ( 0.21 ) |
| Illness | 563 | 4.51 ( 4.15 - 4.9 ) | 4.48 ( 1512.96 ) | 4.45 ( 4.15 ) | 2.16 ( 0.49 ) |
| Balance disorder | 554 | 4.18 ( 3.84 - 4.54 ) | 4.16 ( 1321.04 ) | 4.13 ( 3.85 ) | 2.05 ( 0.38 ) |
| Oropharyngeal pain | 485 | 3.3 ( 3.01 - 3.6 ) | 3.28 ( 766.48 ) | 3.27 ( 3.03 ) | 1.71 ( 0.04 ) |
| Injection site bruising | 457 | 3.95 ( 3.6 - 4.33 ) | 3.94 ( 995.54 ) | 3.92 ( 3.63 ) | 1.97 ( 0.3 ) |
| Device malfunction | 414 | 4.19 ( 3.8 - 4.61 ) | 4.17 ( 992.23 ) | 4.15 ( 3.83 ) | 2.05 ( 0.39 ) |
| Rhinorrhoea | 382 | 3.92 ( 3.55 - 4.34 ) | 3.91 ( 822.37 ) | 3.89 ( 3.57 ) | 1.96 ( 0.29 ) |
| Limb discomfort | 340 | 6.85 ( 6.15 - 7.62 ) | 6.83 ( 1669.61 ) | 6.75 ( 6.17 ) | 2.75 ( 1.09 ) |
| Drug effect less than expected | 312 | 23.06 ( 20.59 - 25.84 ) | 22.99 ( 6288.14 ) | 22.07 ( 20.07 ) | 4.46 ( 2.8 ) |
| Incorrect dose administered by device | 281 | 8.53 ( 7.58 - 9.6 ) | 8.51 ( 1832.25 ) | 8.39 ( 7.6 ) | 3.07 ( 1.4 ) |
| Lower respiratory tract infection | 257 | 3.89 ( 3.44 - 4.4 ) | 3.88 ( 546.89 ) | 3.86 ( 3.49 ) | 1.95 ( 0.28 ) |
| Upper respiratory tract infection | 242 | 3.48 ( 3.07 - 3.96 ) | 3.48 ( 424.83 ) | 3.46 ( 3.11 ) | 1.79 ( 0.13 ) |
| Feeling cold | 240 | 5.83 ( 5.14 - 6.63 ) | 5.82 ( 948.42 ) | 5.77 ( 5.19 ) | 2.53 ( 0.86 ) |
| Therapeutic response shortened | 234 | 6.07 ( 5.33 - 6.9 ) | 6.05 ( 976.21 ) | 6 ( 5.38 ) | 2.58 ( 0.92 ) |
| Wrong technique in device usage process | 231 | 3.54 ( 3.11 - 4.03 ) | 3.54 ( 417.47 ) | 3.52 ( 3.16 ) | 1.81 ( 0.15 ) |
| Temperature intolerance | 216 | 14.32 ( 12.51 - 16.4 ) | 14.29 ( 2600.15 ) | 13.94 ( 12.45 ) | 3.8 ( 2.14 ) |
| Gait inability | 197 | 4.07 ( 3.54 - 4.68 ) | 4.06 ( 451.44 ) | 4.04 ( 3.59 ) | 2.01 ( 0.35 ) |
| Brain fog | 188 | 19.32 ( 16.7 - 22.35 ) | 19.28 ( 3144.16 ) | 18.64 ( 16.5 ) | 4.22 ( 2.55 ) |
| Neuralgia | 149 | 4.04 ( 3.44 - 4.74 ) | 4.03 ( 337.4 ) | 4.01 ( 3.5 ) | 2 ( 0.34 ) |
| Needle issue | 149 | 3.61 ( 3.07 - 4.24 ) | 3.61 ( 278.96 ) | 3.59 ( 3.14 ) | 1.84 ( 0.18 ) |
| Sars-cov-2 test positive | 143 | 6.28 ( 5.32 - 7.4 ) | 6.27 ( 625.74 ) | 6.21 ( 5.4 ) | 2.63 ( 0.97 ) |
| Product supply issue | 134 | 13.18 ( 11.11 - 15.65 ) | 13.17 ( 1469.91 ) | 12.87 ( 11.15 ) | 3.69 ( 2.02 ) |
| Central nervous system lesion | 125 | 5.32 ( 4.46 - 6.35 ) | 5.32 ( 433.92 ) | 5.27 ( 4.55 ) | 2.4 ( 0.73 ) |
| Device defective | 123 | 8.01 ( 6.7 - 9.57 ) | 8 ( 742.34 ) | 7.9 ( 6.8 ) | 2.98 ( 1.31 ) |
| Muscle spasticity | 122 | 6.08 ( 5.09 - 7.27 ) | 6.07 ( 511.31 ) | 6.02 ( 5.18 ) | 2.59 ( 0.92 ) |
| Product temperature excursion issue | 114 | 23.21 ( 19.24 - 28 ) | 23.18 ( 2317.28 ) | 22.24 ( 19.01 ) | 4.48 ( 2.81 ) |
| Incorrect route of product administration | 112 | 4.58 ( 3.8 - 5.52 ) | 4.58 ( 310.34 ) | 4.54 ( 3.89 ) | 2.18 ( 0.52 ) |
| Optic neuritis | 111 | 8.22 ( 6.81 - 9.91 ) | 8.21 ( 691.86 ) | 8.1 ( 6.92 ) | 3.02 ( 1.35 ) |
| Hemiparesis | 108 | 4.35 ( 3.6 - 5.25 ) | 4.34 ( 275.78 ) | 4.32 ( 3.68 ) | 2.11 ( 0.44 ) |
| Lymphocyte count decreased | 101 | 3.58 ( 2.95 - 4.36 ) | 3.58 ( 186.55 ) | 3.56 ( 3.02 ) | 1.83 ( 0.17 ) |
| Muscle tightness | 100 | 4.07 ( 3.34 - 4.95 ) | 4.07 ( 229.47 ) | 4.04 ( 3.43 ) | 2.02 ( 0.35 ) |
| Body temperature increased | 97 | 3.35 ( 2.74 - 4.09 ) | 3.34 ( 158.41 ) | 3.33 ( 2.82 ) | 1.74 ( 0.07 ) |
| Sluggishness | 79 | 5.05 ( 4.05 - 6.31 ) | 5.05 ( 254.26 ) | 5.01 ( 4.16 ) | 2.33 ( 0.66 ) |
| Injection related reaction | 77 | 34.96 ( 27.76 - 44.03 ) | 34.93 ( 2379.91 ) | 32.82 ( 27.06 ) | 5.04 ( 3.37 ) |
| Decreased immune responsiveness | 76 | 5.11 ( 4.07 - 6.4 ) | 5.1 ( 248.43 ) | 5.06 ( 4.19 ) | 2.34 ( 0.67 ) |
| Feeling of body temperature change | 75 | 8.27 ( 6.59 - 10.4 ) | 8.27 ( 471.87 ) | 8.16 ( 6.74 ) | 3.03 ( 1.36 ) |
| Product distribution issue | 69 | 7.82 ( 6.17 - 9.92 ) | 7.82 ( 404.13 ) | 7.72 ( 6.32 ) | 2.95 ( 1.28 ) |
| Band sensation | 68 | 32.74 ( 25.62 - 41.83 ) | 32.71 ( 1968.08 ) | 30.85 ( 25.13 ) | 4.95 ( 3.28 ) |
| Injection site discharge | 63 | 14.76 ( 11.49 - 18.97 ) | 14.75 ( 785.82 ) | 14.38 ( 11.66 ) | 3.85 ( 2.18 ) |
| Injection site extravasation | 63 | 3.25 ( 2.53 - 4.16 ) | 3.25 ( 97.32 ) | 3.23 ( 2.63 ) | 1.69 ( 0.03 ) |
| Injection site discomfort | 60 | 4.07 ( 3.16 - 5.25 ) | 4.07 ( 137.79 ) | 4.04 ( 3.27 ) | 2.02 ( 0.35 ) |
| B-lymphocyte count decreased | 58 | 32.05 ( 24.59 - 41.79 ) | 32.03 ( 1643.66 ) | 30.25 ( 24.23 ) | 4.92 ( 3.25 ) |
| Electric shock sensation | 58 | 19.5 ( 15 - 25.34 ) | 19.49 ( 980.86 ) | 18.83 ( 15.12 ) | 4.23 ( 2.57 ) |
| Peroneal nerve palsy | 58 | 6.64 ( 5.12 - 8.6 ) | 6.64 ( 274.24 ) | 6.57 ( 5.29 ) | 2.72 ( 1.05 ) |
| Expanded disability status scale score increased | 52 | 27.25 ( 20.62 - 36.01 ) | 27.23 ( 1249.31 ) | 25.94 ( 20.54 ) | 4.7 ( 3.03 ) |
| Chronic lymphocytic leukaemia | 49 | 7.08 ( 5.34 - 9.38 ) | 7.07 ( 252.09 ) | 6.99 ( 5.52 ) | 2.81 ( 1.14 ) |
| Neutropenic sepsis | 49 | 4.6 ( 3.47 - 6.09 ) | 4.6 ( 136.75 ) | 4.57 ( 3.61 ) | 2.19 ( 0.52 ) |
| Multiple sclerosis pseudo relapse | 43 | 127.8 ( 91.57 - 178.35 ) | 127.73 ( 4350.15 ) | 102.96 ( 77.91 ) | 6.69 ( 5.01 ) |
| Aspergillus infection | 43 | 4.32 ( 3.2 - 5.84 ) | 4.32 ( 108.92 ) | 4.3 ( 3.34 ) | 2.1 ( 0.44 ) |
| Concomitant disease aggravated | 41 | 3.99 ( 2.94 - 5.43 ) | 3.99 ( 91.29 ) | 3.97 ( 3.07 ) | 1.99 ( 0.32 ) |
| Blood immunoglobulin m decreased | 34 | 25.22 ( 17.88 - 35.59 ) | 25.21 ( 754.47 ) | 24.11 ( 18.07 ) | 4.59 ( 2.92 ) |
| Dysgraphia | 34 | 3.28 ( 2.34 - 4.59 ) | 3.27 ( 53.4 ) | 3.26 ( 2.46 ) | 1.71 ( 0.04 ) |
| Energy increased | 33 | 3.74 ( 2.66 - 5.27 ) | 3.74 ( 65.82 ) | 3.72 ( 2.79 ) | 1.9 ( 0.23 ) |
| Blood immunoglobulin g decreased | 31 | 9.79 ( 6.86 - 13.97 ) | 9.79 ( 240.18 ) | 9.63 ( 7.15 ) | 3.27 ( 1.6 ) |
| Blepharospasm | 31 | 4.04 ( 2.84 - 5.75 ) | 4.04 ( 70.34 ) | 4.02 ( 2.99 ) | 2.01 ( 0.34 ) |
| Trigeminal neuralgia | 30 | 4.9 ( 3.42 - 7.02 ) | 4.9 ( 92.32 ) | 4.87 ( 3.6 ) | 2.28 ( 0.62 ) |
| Jc polyomavirus test positive | 29 | 15.28 ( 10.56 - 22.11 ) | 15.28 ( 376.06 ) | 14.88 ( 10.92 ) | 3.89 ( 2.23 ) |
| Relapsing-remitting multiple sclerosis | 27 | 21.61 ( 14.71 - 31.76 ) | 21.61 ( 509.72 ) | 20.79 ( 15.07 ) | 4.38 ( 2.71 ) |
| Secondary progressive multiple sclerosis | 27 | 8.36 ( 5.72 - 12.23 ) | 8.36 ( 172.23 ) | 8.25 ( 6 ) | 3.04 ( 1.38 ) |
| Muscle fatigue | 24 | 3.91 ( 2.62 - 5.85 ) | 3.91 ( 51.66 ) | 3.89 ( 2.78 ) | 1.96 ( 0.29 ) |
| Relapsing multiple sclerosis | 23 | 48 ( 31.32 - 73.57 ) | 47.99 ( 969.73 ) | 44.06 ( 30.82 ) | 5.46 ( 3.79 ) |
| Monoplegia | 23 | 3.67 ( 2.43 - 5.53 ) | 3.67 ( 44.28 ) | 3.65 ( 2.59 ) | 1.87 ( 0.2 ) |
| Hangover | 22 | 4.17 ( 2.74 - 6.35 ) | 4.17 ( 52.63 ) | 4.15 ( 2.92 ) | 2.05 ( 0.39 ) |
| Clumsiness | 21 | 5.08 ( 3.3 - 7.8 ) | 5.07 ( 68.04 ) | 5.04 ( 3.51 ) | 2.33 ( 0.67 ) |
| Maternal exposure timing unspecified | 21 | 3.92 ( 2.55 - 6.03 ) | 3.92 ( 45.39 ) | 3.9 ( 2.72 ) | 1.96 ( 0.3 ) |
| Immunoglobulins decreased | 20 | 10.78 ( 6.92 - 16.78 ) | 10.77 ( 173.79 ) | 10.58 ( 7.3 ) | 3.4 ( 1.73 ) |
| Maternal exposure during breast feeding | 20 | 3.88 ( 2.5 - 6.02 ) | 3.88 ( 42.37 ) | 3.86 ( 2.67 ) | 1.95 ( 0.28 ) |
| Fine motor skill dysfunction | 19 | 4.16 ( 2.65 - 6.54 ) | 4.16 ( 45.27 ) | 4.14 ( 2.83 ) | 2.05 ( 0.38 ) |
| Exposure to sars-cov-2 | 18 | 6 ( 3.77 - 9.55 ) | 6 ( 74.18 ) | 5.94 ( 4.03 ) | 2.57 ( 0.9 ) |
| Heat exhaustion | 17 | 15.39 ( 9.5 - 24.92 ) | 15.38 ( 222.15 ) | 14.98 ( 10 ) | 3.9 ( 2.23 ) |
| Magnetic resonance imaging abnormal | 17 | 9.9 ( 6.13 - 16 ) | 9.9 ( 133.48 ) | 9.73 ( 6.52 ) | 3.28 ( 1.61 ) |
| Jc virus infection | 17 | 7.46 ( 4.62 - 12.03 ) | 7.45 ( 93.69 ) | 7.36 ( 4.93 ) | 2.88 ( 1.21 ) |
| Lymph node pain | 17 | 6.18 ( 3.83 - 9.97 ) | 6.18 ( 72.98 ) | 6.12 ( 4.1 ) | 2.61 ( 0.95 ) |
| Respiratory symptom | 17 | 4.45 ( 2.76 - 7.17 ) | 4.45 ( 45.08 ) | 4.42 ( 2.96 ) | 2.14 ( 0.48 ) |
| Upper respiratory tract congestion | 17 | 3.65 ( 2.26 - 5.88 ) | 3.65 ( 32.46 ) | 3.63 ( 2.44 ) | 1.86 ( 0.19 ) |
| Progressive multiple sclerosis | 16 | 8.73 ( 5.33 - 14.31 ) | 8.73 ( 107.68 ) | 8.6 ( 5.69 ) | 3.1 ( 1.44 ) |
| Lower respiratory tract congestion | 16 | 5.92 ( 3.62 - 9.69 ) | 5.92 ( 64.7 ) | 5.87 ( 3.88 ) | 2.55 ( 0.88 ) |
| Hemihypoaesthesia | 15 | 40.66 ( 24.05 - 68.76 ) | 40.65 ( 538.54 ) | 37.81 ( 24.36 ) | 5.24 ( 3.56 ) |
| Motion sickness | 15 | 5.45 ( 3.28 - 9.07 ) | 5.45 ( 53.95 ) | 5.4 ( 3.53 ) | 2.43 ( 0.77 ) |
| Injection site hypoaesthesia | 14 | 6.52 ( 3.85 - 11.04 ) | 6.51 ( 64.55 ) | 6.45 ( 4.15 ) | 2.69 ( 1.02 ) |
| Paresis | 13 | 4.16 ( 2.41 - 7.17 ) | 4.16 ( 30.9 ) | 4.13 ( 2.62 ) | 2.05 ( 0.38 ) |
| Bell's palsy | 12 | 6.2 ( 3.51 - 10.96 ) | 6.2 ( 51.77 ) | 6.14 ( 3.82 ) | 2.62 ( 0.95 ) |
| Lhermitte's sign | 11 | 23.23 ( 12.7 - 42.49 ) | 23.23 ( 224.08 ) | 22.29 ( 13.45 ) | 4.48 ( 2.8 ) |
| Blood immunoglobulin a decreased | 11 | 12.94 ( 7.11 - 23.54 ) | 12.94 ( 118.27 ) | 12.65 ( 7.67 ) | 3.66 ( 1.99 ) |
| Mental fatigue | 11 | 5.29 ( 2.92 - 9.58 ) | 5.29 ( 37.86 ) | 5.24 ( 3.19 ) | 2.39 ( 0.72 ) |
| Injection site paraesthesia | 11 | 5.21 ( 2.87 - 9.43 ) | 5.21 ( 37.01 ) | 5.16 ( 3.14 ) | 2.37 ( 0.7 ) |
| Product dispensing issue | 11 | 3.86 ( 2.13 - 6.98 ) | 3.86 ( 23.12 ) | 3.84 ( 2.34 ) | 1.94 ( 0.27 ) |
| Device dispensing error | 10 | 6.57 ( 3.52 - 12.26 ) | 6.57 ( 46.66 ) | 6.5 ( 3.86 ) | 2.7 ( 1.03 ) |
| Bladder spasm | 10 | 3.95 ( 2.12 - 7.36 ) | 3.95 ( 21.87 ) | 3.93 ( 2.33 ) | 1.97 ( 0.31 ) |
| Body temperature abnormal | 10 | 3.52 ( 1.89 - 6.56 ) | 3.52 ( 17.92 ) | 3.5 ( 2.08 ) | 1.81 ( 0.14 ) |
| Diplegia | 10 | 3.48 ( 1.87 - 6.47 ) | 3.48 ( 17.51 ) | 3.46 ( 2.06 ) | 1.79 ( 0.12 ) |
| Expanded disability status scale | 9 | 77.58 ( 38.53 - 156.22 ) | 77.58 ( 592.83 ) | 67.73 ( 37.71 ) | 6.08 ( 4.38 ) |
| Cd19 lymphocytes decreased | 8 | 56.09 ( 27.06 - 116.28 ) | 56.08 ( 391.1 ) | 50.78 ( 27.59 ) | 5.67 ( 3.97 ) |
| T-lymphocyte count decreased | 8 | 8.06 ( 4.01 - 16.2 ) | 8.06 ( 48.71 ) | 7.95 ( 4.43 ) | 2.99 ( 1.32 ) |
| Paternal exposure during pregnancy | 8 | 6.56 ( 3.27 - 13.18 ) | 6.56 ( 37.25 ) | 6.49 ( 3.62 ) | 2.7 ( 1.03 ) |
| Groin abscess | 8 | 4.49 ( 2.24 - 9 ) | 4.49 ( 21.51 ) | 4.46 ( 2.49 ) | 2.16 ( 0.49 ) |
| Optic nerve disorder | 8 | 3.77 ( 1.88 - 7.56 ) | 3.77 ( 16.18 ) | 3.75 ( 2.1 ) | 1.91 ( 0.24 ) |
| B-cell aplasia | 7 | 24.22 ( 11.35 - 51.66 ) | 24.21 ( 148.93 ) | 23.19 ( 12.3 ) | 4.54 ( 2.85 ) |
| Ophthalmic migraine | 7 | 10.4 ( 4.92 - 21.97 ) | 10.4 ( 58.3 ) | 10.21 ( 5.46 ) | 3.35 ( 1.68 ) |
| Muscle discomfort | 7 | 6.45 ( 3.06 - 13.58 ) | 6.45 ( 31.82 ) | 6.38 ( 3.42 ) | 2.67 ( 1 ) |
| Myelin oligodendrocyte glycoprotein antibody-associated disease | 6 | 50.89 ( 22.01 - 117.64 ) | 50.88 ( 267.53 ) | 46.48 ( 23.05 ) | 5.54 ( 3.84 ) |
| B-lymphocyte count increased | 6 | 19.97 ( 8.84 - 45.12 ) | 19.97 ( 104.15 ) | 19.27 ( 9.74 ) | 4.27 ( 2.59 ) |
| Hemiparaesthesia | 6 | 9.8 ( 4.37 - 21.97 ) | 9.8 ( 46.53 ) | 9.64 ( 4.9 ) | 3.27 ( 1.6 ) |
| Norovirus infection | 6 | 5.86 ( 2.62 - 13.11 ) | 5.86 ( 23.94 ) | 5.81 ( 2.96 ) | 2.54 ( 0.87 ) |
| Altered visual depth perception | 6 | 5.22 ( 2.34 - 11.67 ) | 5.22 ( 20.29 ) | 5.18 ( 2.64 ) | 2.37 ( 0.7 ) |
| Richter's syndrome | 6 | 4.33 ( 1.94 - 9.68 ) | 4.33 ( 15.26 ) | 4.31 ( 2.2 ) | 2.11 ( 0.44 ) |
| Piloerection | 6 | 3.97 ( 1.78 - 8.86 ) | 3.97 ( 13.22 ) | 3.95 ( 2.02 ) | 1.98 ( 0.31 ) |
| Progressive relapsing multiple sclerosis | 5 | 27.67 ( 11.26 - 68.02 ) | 27.67 ( 122.12 ) | 26.34 ( 12.41 ) | 4.72 ( 3.03 ) |
| Exposure to extreme temperature | 5 | 20.38 ( 8.34 - 49.8 ) | 20.38 ( 88.71 ) | 19.66 ( 9.31 ) | 4.3 ( 2.61 ) |
| Immunoglobulins abnormal | 5 | 13.91 ( 5.72 - 33.81 ) | 13.91 ( 58.36 ) | 13.58 ( 6.46 ) | 3.76 ( 2.08 ) |
| Lipoedema | 5 | 10.11 ( 4.17 - 24.5 ) | 10.11 ( 40.28 ) | 9.94 ( 4.74 ) | 3.31 ( 1.64 ) |
| Muscle strength abnormal | 5 | 8.48 ( 3.51 - 20.52 ) | 8.48 ( 32.47 ) | 8.36 ( 3.99 ) | 3.06 ( 1.39 ) |
| Facial discomfort | 5 | 6.81 ( 2.82 - 16.46 ) | 6.81 ( 24.47 ) | 6.74 ( 3.22 ) | 2.75 ( 1.08 ) |
| Electric shock | 5 | 4.27 ( 1.77 - 10.31 ) | 4.27 ( 12.44 ) | 4.25 ( 2.03 ) | 2.09 ( 0.42 ) |
| Device temperature issue | 4 | 47.8 ( 17.18 - 133.04 ) | 47.8 ( 168.01 ) | 43.9 ( 18.64 ) | 5.46 ( 3.74 ) |
| B-lymphocyte count abnormal | 4 | 22.86 ( 8.4 - 62.21 ) | 22.86 ( 80.13 ) | 21.95 ( 9.5 ) | 4.46 ( 2.76 ) |
| Polycystic ovarian syndrome | 4 | 20.62 ( 7.59 - 56 ) | 20.62 ( 71.85 ) | 19.88 ( 8.62 ) | 4.31 ( 2.62 ) |
| Sensory overload | 4 | 17.38 ( 6.42 - 47.07 ) | 17.38 ( 59.78 ) | 16.86 ( 7.33 ) | 4.08 ( 2.39 ) |
| Tumefactive multiple sclerosis | 4 | 15.24 ( 5.64 - 41.19 ) | 15.24 ( 51.72 ) | 14.84 ( 6.46 ) | 3.89 ( 2.21 ) |
| Herpes zoster reactivation | 4 | 10.84 ( 4.03 - 29.18 ) | 10.84 ( 35.01 ) | 10.64 ( 4.65 ) | 3.41 ( 1.73 ) |
| Fungal foot infection | 4 | 8.03 ( 2.99 - 21.55 ) | 8.03 ( 24.24 ) | 7.92 ( 3.47 ) | 2.99 ( 1.31 ) |
| Graves' disease | 4 | 7.03 ( 2.62 - 18.87 ) | 7.03 ( 20.43 ) | 6.95 ( 3.05 ) | 2.8 ( 1.12 ) |
| Decreased vibratory sense | 4 | 6.85 ( 2.55 - 18.37 ) | 6.85 ( 19.73 ) | 6.78 ( 2.97 ) | 2.76 ( 1.09 ) |
| Vibration syndrome | 3 | 87.63 ( 25.81 - 297.52 ) | 87.63 ( 220.23 ) | 75.26 ( 27.06 ) | 6.23 ( 4.46 ) |
| Injection site muscle weakness | 3 | 54.39 ( 16.57 - 178.57 ) | 54.39 ( 142.49 ) | 49.39 ( 18.27 ) | 5.63 ( 3.89 ) |
| Alexia | 3 | 28.68 ( 8.97 - 91.68 ) | 28.68 ( 76 ) | 27.25 ( 10.3 ) | 4.77 ( 3.06 ) |
| Administration site wound | 3 | 26.74 ( 8.38 - 85.29 ) | 26.74 ( 70.72 ) | 25.49 ( 9.66 ) | 4.67 ( 2.97 ) |
| Blood immunoglobulin g abnormal | 3 | 18.78 ( 5.94 - 59.41 ) | 18.78 ( 48.75 ) | 18.17 ( 6.93 ) | 4.18 ( 2.49 ) |
| Supportive care | 3 | 18.13 ( 5.74 - 57.32 ) | 18.13 ( 46.94 ) | 17.56 ( 6.7 ) | 4.13 ( 2.44 ) |
| Fumbling | 3 | 16.96 ( 5.37 - 53.55 ) | 16.96 ( 43.65 ) | 16.46 ( 6.29 ) | 4.04 ( 2.35 ) |
| Cytokine increased | 3 | 14.74 ( 4.68 - 46.44 ) | 14.74 ( 37.38 ) | 14.37 ( 5.5 ) | 3.84 ( 2.16 ) |
| Loss of bladder sensation | 3 | 11.77 ( 3.75 - 36.96 ) | 11.77 ( 28.92 ) | 11.54 ( 4.43 ) | 3.53 ( 1.84 ) |
| T-lymphocyte count increased | 3 | 10.95 ( 3.49 - 34.37 ) | 10.95 ( 26.58 ) | 10.75 ( 4.13 ) | 3.43 ( 1.74 ) |
| B-cell small lymphocytic lymphoma | 3 | 10.38 ( 3.31 - 32.54 ) | 10.38 ( 24.93 ) | 10.2 ( 3.92 ) | 3.35 ( 1.67 ) |
| Pseudostroke | 3 | 9.07 ( 2.9 - 28.38 ) | 9.07 ( 21.16 ) | 8.93 ( 3.44 ) | 3.16 ( 1.48 ) |
| Neurogenic bowel | 3 | 9.01 ( 2.88 - 28.22 ) | 9.01 ( 21.01 ) | 8.88 ( 3.42 ) | 3.15 ( 1.47 ) |
| Squamous cell carcinoma of head and neck | 3 | 8.05 ( 2.57 - 25.17 ) | 8.05 ( 18.24 ) | 7.94 ( 3.06 ) | 2.99 ( 1.31 ) |
| Babesiosis | 3 | 7.62 ( 2.44 - 23.82 ) | 7.62 ( 17.01 ) | 7.53 ( 2.9 ) | 2.91 ( 1.23 ) |
| Craniofacial fracture | 3 | 6.14 ( 1.97 - 19.16 ) | 6.14 ( 12.75 ) | 6.08 ( 2.35 ) | 2.6 ( 0.93 ) |
| Acute hepatitis b | 3 | 5.89 ( 1.89 - 18.37 ) | 5.89 ( 12.03 ) | 5.83 ( 2.25 ) | 2.54 ( 0.87 ) |
| Central nervous system inflammation | 3 | 5.8 ( 1.86 - 18.09 ) | 5.8 ( 11.78 ) | 5.75 ( 2.22 ) | 2.52 ( 0.85 ) |
| Polymerase chain reaction positive | 3 | 5.46 ( 1.75 - 17.02 ) | 5.46 ( 10.81 ) | 5.41 ( 2.09 ) | 2.44 ( 0.76 ) |

Abbreviation: ROR, reporting odds ratio; PRR, proportional reporting ratio; EBGM, empirical Bayesian geometric mean; EBGM05, the lower limit of the 95% CI of EBGM; IC, information component; IC025, the lower limit of the 95% CI of the IC; CI, confidence interval; PT,preferred term.

**Supplementary Table 4:** Top 50 most frequent adverse events for Ofatumumab at the preferred term (PT) level in males from FAERS data

| PT | Case numbers | ROR(95%CI) | PRR(χ^2^) | EBGM(EBGM05) | IC(IC025) |
| --- | --- | --- | --- | --- | --- |
| Fatigue | 882 | 3.76 ( 3.51 - 4.02 ) | 3.63 ( 1695.76 ) | 3.62 ( 3.42 ) | 1.86 ( 0.19 ) |
| Chills | 765 | 20.84 ( 19.37 - 22.42 ) | 20.07 ( 13551.26 ) | 19.61 ( 18.44 ) | 4.29 ( 2.63 ) |
| Pyrexia | 758 | 6.21 ( 5.77 - 6.68 ) | 6.01 ( 3162.64 ) | 5.97 ( 5.62 ) | 2.58 ( 0.91 ) |
| Headache | 642 | 4.34 ( 4.01 - 4.7 ) | 4.23 ( 1587.66 ) | 4.21 ( 3.94 ) | 2.07 ( 0.41 ) |
| Influenza like illness | 573 | 25.76 ( 23.68 - 28.03 ) | 25.04 ( 12844.6 ) | 24.32 ( 22.66 ) | 4.6 ( 2.94 ) |
| Pain | 517 | 2.83 ( 2.6 - 3.09 ) | 2.78 ( 594.86 ) | 2.78 ( 2.58 ) | 1.47 ( -0.19 ) |
| Asthenia | 291 | 2.27 ( 2.02 - 2.54 ) | 2.25 ( 202.12 ) | 2.24 ( 2.04 ) | 1.17 ( -0.5 ) |
| Gait disturbance | 269 | 4.43 ( 3.92 - 4.99 ) | 4.38 ( 700.13 ) | 4.36 ( 3.94 ) | 2.13 ( 0.46 ) |
| Drug ineffective | 267 | 0.64 ( 0.57 - 0.73 ) | 0.65 ( 51.67 ) | 0.65 ( 0.59 ) | -0.62 ( -2.29 ) |
| Hypoaesthesia | 241 | 6.02 ( 5.3 - 6.84 ) | 5.96 ( 988.55 ) | 5.92 ( 5.32 ) | 2.57 ( 0.9 ) |
| Covid-19 | 233 | 3.79 ( 3.33 - 4.32 ) | 3.76 ( 471.27 ) | 3.75 ( 3.36 ) | 1.91 ( 0.24 ) |
| Feeling abnormal | 232 | 3.52 ( 3.09 - 4.01 ) | 3.49 ( 411.83 ) | 3.48 ( 3.12 ) | 1.8 ( 0.13 ) |
| Multiple sclerosis relapse | 221 | 14.31 ( 12.52 - 16.36 ) | 14.16 ( 2658.36 ) | 13.93 ( 12.46 ) | 3.8 ( 2.13 ) |
| Dizziness | 216 | 1.51 ( 1.32 - 1.72 ) | 1.5 ( 36.32 ) | 1.5 ( 1.34 ) | 0.59 ( -1.08 ) |
| Nausea | 202 | 1.08 ( 0.94 - 1.25 ) | 1.08 ( 1.32 ) | 1.08 ( 0.97 ) | 0.12 ( -1.55 ) |
| Malaise | 194 | 1.53 ( 1.33 - 1.76 ) | 1.52 ( 35.03 ) | 1.52 ( 1.35 ) | 0.61 ( -1.06 ) |
| Pneumonia | 164 | 1.32 ( 1.13 - 1.54 ) | 1.32 ( 12.77 ) | 1.32 ( 1.16 ) | 0.4 ( -1.27 ) |
| Fall | 164 | 1.59 ( 1.36 - 1.86 ) | 1.59 ( 35.71 ) | 1.59 ( 1.39 ) | 0.67 ( -1 ) |
| Myalgia | 159 | 3.08 ( 2.63 - 3.6 ) | 3.06 ( 220.05 ) | 3.05 ( 2.68 ) | 1.61 ( -0.06 ) |
| Muscular weakness | 156 | 4.19 ( 3.58 - 4.91 ) | 4.17 ( 374.22 ) | 4.15 ( 3.64 ) | 2.05 ( 0.39 ) |
| Inappropriate schedule of product administration | 156 | 3.07 ( 2.62 - 3.6 ) | 3.05 ( 215.26 ) | 3.05 ( 2.67 ) | 1.61 ( -0.06 ) |
| Muscle spasms | 153 | 3 ( 2.56 - 3.52 ) | 2.98 ( 201.45 ) | 2.98 ( 2.6 ) | 1.57 ( -0.09 ) |
| Nasopharyngitis | 149 | 2.96 ( 2.52 - 3.47 ) | 2.94 ( 190.69 ) | 2.93 ( 2.56 ) | 1.55 ( -0.11 ) |
| Accidental exposure to product | 147 | 5.47 ( 4.65 - 6.44 ) | 5.44 ( 530.07 ) | 5.41 ( 4.72 ) | 2.44 ( 0.77 ) |
| Balance disorder | 145 | 5.21 ( 4.42 - 6.14 ) | 5.18 ( 486.51 ) | 5.15 ( 4.49 ) | 2.37 ( 0.7 ) |
| Product dose omission issue | 143 | 1.92 ( 1.63 - 2.27 ) | 1.92 ( 62.9 ) | 1.92 ( 1.67 ) | 0.94 ( -0.73 ) |
| Tremor | 142 | 2.88 ( 2.44 - 3.4 ) | 2.87 ( 172.4 ) | 2.86 ( 2.49 ) | 1.52 ( -0.15 ) |
| Vomiting | 140 | 1.16 ( 0.98 - 1.37 ) | 1.16 ( 3.05 ) | 1.16 ( 1.01 ) | 0.21 ( -1.45 ) |
| Arthralgia | 139 | 1.27 ( 1.08 - 1.5 ) | 1.27 ( 8.06 ) | 1.27 ( 1.1 ) | 0.35 ( -1.32 ) |
| Back pain | 137 | 2.02 ( 1.71 - 2.39 ) | 2.01 ( 69.74 ) | 2.01 ( 1.75 ) | 1.01 ( -0.66 ) |
| Pain in extremity | 137 | 1.73 ( 1.46 - 2.04 ) | 1.72 ( 41.37 ) | 1.72 ( 1.49 ) | 0.78 ( -0.88 ) |
| Injection site pain | 129 | 1.79 ( 1.51 - 2.13 ) | 1.79 ( 44.63 ) | 1.78 ( 1.54 ) | 0.83 ( -0.83 ) |
| Cough | 127 | 1.48 ( 1.24 - 1.76 ) | 1.47 ( 19.32 ) | 1.47 ( 1.27 ) | 0.56 ( -1.11 ) |
| Paraesthesia | 124 | 2.99 ( 2.5 - 3.56 ) | 2.97 ( 162.07 ) | 2.97 ( 2.56 ) | 1.57 ( -0.1 ) |
| Dyspnoea | 112 | 0.6 ( 0.5 - 0.72 ) | 0.6 ( 29.51 ) | 0.6 ( 0.52 ) | -0.73 ( -2.39 ) |
| Febrile neutropenia | 111 | 4.14 ( 3.44 - 5 ) | 4.13 ( 262 ) | 4.11 ( 3.52 ) | 2.04 ( 0.37 ) |
| Insomnia | 110 | 1.34 ( 1.11 - 1.61 ) | 1.33 ( 9.21 ) | 1.33 ( 1.14 ) | 0.41 ( -1.25 ) |
| Memory impairment | 101 | 2.43 ( 2 - 2.96 ) | 2.43 ( 84.57 ) | 2.42 ( 2.06 ) | 1.28 ( -0.39 ) |
| Rash | 98 | 0.75 ( 0.61 - 0.91 ) | 0.75 ( 8.27 ) | 0.75 ( 0.63 ) | -0.42 ( -2.08 ) |
| Diarrhoea | 97 | 0.47 ( 0.38 - 0.57 ) | 0.47 ( 58.18 ) | 0.47 ( 0.4 ) | -1.08 ( -2.75 ) |
| Illness | 93 | 4.75 ( 3.87 - 5.83 ) | 4.73 ( 272.42 ) | 4.71 ( 3.97 ) | 2.24 ( 0.57 ) |
| Device malfunction | 91 | 4.71 ( 3.83 - 5.79 ) | 4.69 ( 262.98 ) | 4.67 ( 3.93 ) | 2.22 ( 0.56 ) |
| Mobility decreased | 91 | 4.33 ( 3.52 - 5.32 ) | 4.31 ( 230.34 ) | 4.29 ( 3.61 ) | 2.1 ( 0.44 ) |
| Hyperhidrosis | 90 | 2.05 ( 1.66 - 2.52 ) | 2.04 ( 47.79 ) | 2.04 ( 1.71 ) | 1.03 ( -0.64 ) |
| Death | 89 | 0.22 ( 0.18 - 0.27 ) | 0.23 ( 239.66 ) | 0.23 ( 0.19 ) | -2.14 ( -3.81 ) |
| Condition aggravated | 86 | 0.98 ( 0.79 - 1.21 ) | 0.98 ( 0.03 ) | 0.98 ( 0.82 ) | -0.03 ( -1.69 ) |
| Drug effect less than expected | 84 | 34.25 ( 27.52 - 42.63 ) | 34.11 ( 2591.04 ) | 32.77 ( 27.29 ) | 5.03 ( 3.37 ) |
| Urinary tract infection | 82 | 2.28 ( 1.83 - 2.83 ) | 2.27 ( 58.19 ) | 2.27 ( 1.89 ) | 1.18 ( -0.49 ) |
| Pruritus | 79 | 0.78 ( 0.63 - 0.98 ) | 0.78 ( 4.68 ) | 0.79 ( 0.65 ) | -0.35 ( -2.02 ) |
| Rhinorrhoea | 78 | 3.94 ( 3.15 - 4.92 ) | 3.92 ( 169.3 ) | 3.91 ( 3.24 ) | 1.97 ( 0.3 ) |

Abbreviation: Asterisks (*) indicate statistically significant signals in algorithm; ROR, reporting odds ratio; PRR, proportional reporting ratio; EBGM, empirical Bayesian geometric mean; EBGM05, the lower limit of the 95% CI of EBGM; IC, information component; IC025, the lower limit of the 95% CI of the IC; CI, confidence interval; PT,preferred term; AEs, adverse events.

**Supplementary Table 5**: Top 50 most frequent adverse events for Ofatumumab at the PT level in females from FAERS data

| PT | Case numbers | ROR(95%CI) | PRR(χ^2^) | EBGM(EBGM05) | IC(IC025) |
| --- | --- | --- | --- | --- | --- |
| Fatigue | 3421 | 3.84 ( 3.71 - 3.98 ) | 3.7 ( 6765.6 ) | 3.67 ( 3.57 ) | 1.88 ( 0.21 ) |
| Headache | 3129 | 3.94 ( 3.8 - 4.09 ) | 3.8 ( 6488.67 ) | 3.78 ( 3.67 ) | 1.92 ( 0.25 ) |
| Pain | 2293 | 2.92 ( 2.8 - 3.05 ) | 2.86 ( 2782.53 ) | 2.84 ( 2.75 ) | 1.51 ( -0.16 ) |
| Chills | 2193 | 18.1 ( 17.33 - 18.91 ) | 17.54 ( 32846.31 ) | 16.85 ( 16.25 ) | 4.07 ( 2.41 ) |
| Pyrexia | 2068 | 6.13 ( 5.87 - 6.41 ) | 5.97 ( 8482.85 ) | 5.9 ( 5.69 ) | 2.56 ( 0.89 ) |
| Influenza like illness | 1741 | 17.12 ( 16.31 - 17.97 ) | 16.7 ( 24718.22 ) | 16.08 ( 15.44 ) | 4.01 ( 2.34 ) |
| Nausea | 1198 | 1.21 ( 1.14 - 1.28 ) | 1.2 ( 41.14 ) | 1.2 ( 1.15 ) | 0.26 ( -1.4 ) |
| Covid-19 | 951 | 4.6 ( 4.31 - 4.9 ) | 4.55 ( 2609.06 ) | 4.51 ( 4.27 ) | 2.17 ( 0.51 ) |
| Asthenia | 812 | 2 ( 1.87 - 2.15 ) | 1.99 ( 400.02 ) | 1.98 ( 1.87 ) | 0.99 ( -0.68 ) |
| Multiple sclerosis relapse | 800 | 7.13 ( 6.65 - 7.65 ) | 7.06 ( 4094.16 ) | 6.95 ( 6.56 ) | 2.8 ( 1.13 ) |
| Hypoaesthesia | 775 | 4.1 ( 3.82 - 4.4 ) | 4.06 ( 1777.81 ) | 4.03 ( 3.8 ) | 2.01 ( 0.35 ) |
| Feeling abnormal | 761 | 2.47 ( 2.3 - 2.66 ) | 2.46 ( 656.27 ) | 2.45 ( 2.31 ) | 1.29 ( -0.37 ) |
| Malaise | 742 | 1.28 ( 1.19 - 1.37 ) | 1.28 ( 44.3 ) | 1.27 ( 1.2 ) | 0.35 ( -1.32 ) |
| Dizziness | 740 | 1.27 ( 1.18 - 1.37 ) | 1.27 ( 42.11 ) | 1.27 ( 1.19 ) | 0.34 ( -1.32 ) |
| Gait disturbance | 736 | 2.98 ( 2.77 - 3.2 ) | 2.96 ( 949.66 ) | 2.94 ( 2.77 ) | 1.56 ( -0.11 ) |
| Pain in extremity | 720 | 1.8 ( 1.67 - 1.94 ) | 1.79 ( 251.6 ) | 1.79 ( 1.68 ) | 0.84 ( -0.83 ) |
| Nasopharyngitis | 685 | 2.83 ( 2.63 - 3.06 ) | 2.82 ( 799.14 ) | 2.8 ( 2.63 ) | 1.49 ( -0.18 ) |
| Injection site pain | 662 | 1.73 ( 1.61 - 1.87 ) | 1.73 ( 202.74 ) | 1.72 ( 1.62 ) | 0.79 ( -0.88 ) |
| Drug ineffective | 606 | 0.41 ( 0.38 - 0.45 ) | 0.42 ( 499.05 ) | 0.42 ( 0.39 ) | -1.25 ( -2.92 ) |
| Arthralgia | 579 | 1.09 ( 1.01 - 1.19 ) | 1.09 ( 4.49 ) | 1.09 ( 1.02 ) | 0.13 ( -1.54 ) |
| Back pain | 557 | 1.92 ( 1.76 - 2.09 ) | 1.91 ( 241.6 ) | 1.91 ( 1.78 ) | 0.93 ( -0.74 ) |
| Myalgia | 553 | 2.95 ( 2.71 - 3.21 ) | 2.93 ( 701.48 ) | 2.92 ( 2.72 ) | 1.55 ( -0.12 ) |
| Inappropriate schedule of product administration | 553 | 3.29 ( 3.03 - 3.58 ) | 3.27 ( 868.44 ) | 3.26 ( 3.03 ) | 1.7 ( 0.04 ) |
| Accidental exposure to product | 536 | 4.96 ( 4.55 - 5.4 ) | 4.92 ( 1658.66 ) | 4.88 ( 4.54 ) | 2.29 ( 0.62 ) |
| Cough | 534 | 1.61 ( 1.48 - 1.75 ) | 1.6 ( 121.77 ) | 1.6 ( 1.49 ) | 0.68 ( -0.99 ) |
| Vomiting | 525 | 0.95 ( 0.87 - 1.03 ) | 0.95 ( 1.55 ) | 0.95 ( 0.88 ) | -0.08 ( -1.74 ) |
| Urinary tract infection | 513 | 2.15 ( 1.97 - 2.34 ) | 2.14 ( 310.88 ) | 2.13 ( 1.98 ) | 1.09 ( -0.57 ) |
| Fall | 507 | 1.25 ( 1.15 - 1.37 ) | 1.25 ( 25.51 ) | 1.25 ( 1.16 ) | 0.32 ( -1.34 ) |
| Product dose omission issue | 468 | 1.83 ( 1.67 - 2 ) | 1.82 ( 172.92 ) | 1.82 ( 1.68 ) | 0.86 ( -0.8 ) |
| Paraesthesia | 464 | 2.32 ( 2.12 - 2.55 ) | 2.32 ( 345.81 ) | 2.31 ( 2.14 ) | 1.21 ( -0.46 ) |
| Muscle spasms | 454 | 1.95 ( 1.78 - 2.14 ) | 1.94 ( 206.84 ) | 1.94 ( 1.79 ) | 0.95 ( -0.71 ) |
| Muscular weakness | 444 | 3.36 ( 3.06 - 3.69 ) | 3.35 ( 726.37 ) | 3.33 ( 3.08 ) | 1.73 ( 0.07 ) |
| Illness | 434 | 4.15 ( 3.77 - 4.56 ) | 4.13 ( 1019.7 ) | 4.1 ( 3.78 ) | 2.03 ( 0.37 ) |
| Tremor | 429 | 2.27 ( 2.06 - 2.5 ) | 2.26 ( 301.47 ) | 2.26 ( 2.08 ) | 1.17 ( -0.49 ) |
| Balance disorder | 385 | 3.65 ( 3.3 - 4.04 ) | 3.64 ( 730.98 ) | 3.61 ( 3.32 ) | 1.85 ( 0.19 ) |
| Injection site bruising | 385 | 3.31 ( 2.99 - 3.66 ) | 3.3 ( 612.57 ) | 3.28 ( 3.01 ) | 1.71 ( 0.05 ) |
| Memory impairment | 383 | 2.11 ( 1.9 - 2.33 ) | 2.1 ( 220.18 ) | 2.09 ( 1.93 ) | 1.07 ( -0.6 ) |
| Oropharyngeal pain | 380 | 2.99 ( 2.7 - 3.31 ) | 2.98 ( 497.59 ) | 2.97 ( 2.73 ) | 1.57 ( -0.1 ) |
| Migraine | 378 | 2.7 ( 2.44 - 2.98 ) | 2.69 ( 398.41 ) | 2.68 ( 2.46 ) | 1.42 ( -0.25 ) |
| Diarrhoea | 326 | 0.44 ( 0.4 - 0.49 ) | 0.44 ( 230.04 ) | 0.44 ( 0.41 ) | -1.17 ( -2.84 ) |
| Pruritus | 320 | 0.75 ( 0.67 - 0.84 ) | 0.75 ( 26.49 ) | 0.75 ( 0.69 ) | -0.41 ( -2.08 ) |
| Insomnia | 318 | 1.03 ( 0.93 - 1.16 ) | 1.03 ( 0.36 ) | 1.03 ( 0.94 ) | 0.05 ( -1.62 ) |
| Device malfunction | 312 | 4.36 ( 3.9 - 4.88 ) | 4.35 ( 796 ) | 4.31 ( 3.93 ) | 2.11 ( 0.44 ) |
| Dyspnoea | 302 | 0.47 ( 0.42 - 0.53 ) | 0.47 ( 178.45 ) | 0.47 ( 0.43 ) | -1.08 ( -2.74 ) |
| Rash | 298 | 0.62 ( 0.55 - 0.69 ) | 0.62 ( 70.99 ) | 0.62 ( 0.56 ) | -0.69 ( -2.36 ) |
| Rhinorrhoea | 294 | 3.82 ( 3.41 - 4.29 ) | 3.81 ( 604.37 ) | 3.78 ( 3.44 ) | 1.92 ( 0.25 ) |
| Alopecia | 286 | 0.92 ( 0.82 - 1.03 ) | 0.92 ( 2.02 ) | 0.92 ( 0.83 ) | -0.12 ( -1.79 ) |
| Condition aggravated | 280 | 0.92 ( 0.82 - 1.04 ) | 0.92 ( 1.88 ) | 0.92 ( 0.84 ) | -0.12 ( -1.78 ) |
| Injection site haemorrhage | 279 | 2.9 ( 2.58 - 3.26 ) | 2.89 ( 343.52 ) | 2.88 ( 2.61 ) | 1.53 ( -0.14 ) |
| Stress | 262 | 2.61 ( 2.31 - 2.95 ) | 2.61 ( 258.07 ) | 2.6 ( 2.34 ) | 1.38 ( -0.29 ) |

Abbreviation: Asterisks (*) indicate statistically significant signals in algorithm; ROR, reporting odds ratio; PRR, proportional reporting ratio; EBGM, empirical Bayesian geometric mean; EBGM05, the lower limit of the 95% CI of EBGM; IC, information component; IC025, the lower limit of the 95% CI of the IC; CI, confidence interval; PT,preferred term; AEs, adverse events.

**Supplementary Table 6**: Adverse events at the PT level for Ofatumumab in patients aged under 18 from FAERS data

| PT | Case numbers | ROR(95%CI) | PRR(χ^2^) | EBGM(EBGM05) | IC(IC025) |
| --- | --- | --- | --- | --- | --- |
| Product use in unapproved indication | 13 | 11.9 ( 6.74 - 21.02 ) | 10.96 ( 118.44 ) | 10.95 ( 6.8 ) | 3.45 ( 1.77 ) |
| Nephrotic syndrome | 7 | 147.66 ( 68.83 - 316.79 ) | 140.81 ( 959.82 ) | 139.05 ( 73.42 ) | 7.12 ( 5.43 ) |
| Off label use | 5 | 1.19 ( 0.49 - 2.89 ) | 1.18 ( 0.14 ) | 1.18 ( 0.56 ) | 0.24 ( -1.44 ) |
| Urticaria | 4 | 6.28 ( 2.32 - 16.96 ) | 6.14 ( 17.27 ) | 6.13 ( 2.67 ) | 2.62 ( 0.93 ) |
| Chills | 4 | 24.57 ( 9.09 - 66.41 ) | 23.94 ( 87.83 ) | 23.89 ( 10.4 ) | 4.58 ( 2.89 ) |
| Nausea | 4 | 3.76 ( 1.39 - 10.15 ) | 3.68 ( 7.87 ) | 3.68 ( 1.6 ) | 1.88 ( 0.2 ) |
| Pyrexia | 3 | 1.6 ( 0.51 - 5.03 ) | 1.59 ( 0.67 ) | 1.59 ( 0.61 ) | 0.67 ( -1.01 ) |
| Blood immunoglobulin a decreased | 3 | 636.7 ( 196.75 - 2060.43 ) | 623.99 ( 1766.06 ) | 590.61 ( 221.08 ) | 9.21 ( 7.48 ) |
| Throat tightness | 3 | 56.6 ( 18 - 178.03 ) | 55.49 ( 159.77 ) | 55.22 ( 21.17 ) | 5.79 ( 4.1 ) |
| Dyspnoea | 3 | 3.92 ( 1.25 - 12.31 ) | 3.86 ( 6.4 ) | 3.86 ( 1.48 ) | 1.95 ( 0.27 ) |
| Hypersensitivity | 3 | 5.86 ( 1.87 - 18.39 ) | 5.76 ( 11.85 ) | 5.76 ( 2.21 ) | 2.53 ( 0.84 ) |
| Product use issue | 3 | 2.18 ( 0.7 - 6.84 ) | 2.16 ( 1.88 ) | 2.16 ( 0.83 ) | 1.11 ( -0.57 ) |
| pain | 3 | 4.92 ( 1.57 - 15.44 ) | 4.84 ( 9.19 ) | 4.84 ( 1.86 ) | 2.28 ( 0.59 ) |
| foetal exposure during pregnancy | 3 | 5.45 ( 1.74 - 17.1 ) | 5.36 ( 10.67 ) | 5.36 ( 2.06 ) | 2.42 ( 0.74 ) |
| anaphylactic reaction | 2 | 7.94 ( 1.97 - 32.04 ) | 7.84 ( 11.95 ) | 7.84 ( 2.44 ) | 2.97 ( 1.29 ) |
| cyanosis | 2 | 12.96 ( 3.21 - 52.36 ) | 12.8 ( 21.76 ) | 12.79 ( 3.98 ) | 3.68 ( 1.99 ) |
| infusion related reaction | 2 | 9 ( 2.23 - 36.36 ) | 8.9 ( 14.03 ) | 8.89 ( 2.77 ) | 3.15 ( 1.47 ) |
| rash | 2 | 1.49 ( 0.37 - 5.99 ) | 1.48 ( 0.31 ) | 1.48 ( 0.46 ) | 0.56 ( -1.12 ) |
| dizziness | 2 | 3.64 ( 0.9 - 14.71 ) | 3.61 ( 3.78 ) | 3.61 ( 1.12 ) | 1.85 ( 0.17 ) |
| influenza like illness | 2 | 29.12 ( 7.2 - 117.74 ) | 28.75 ( 53.45 ) | 28.67 ( 8.91 ) | 4.84 ( 3.16 ) |
| vomiting | 2 | 1.06 ( 0.26 - 4.26 ) | 1.05 ( 0.01 ) | 1.05 ( 0.33 ) | 0.08 ( -1.61 ) |
| accidental exposure to product by child | 2 | 4.39 ( 1.09 - 17.72 ) | 4.35 ( 5.16 ) | 4.34 ( 1.35 ) | 2.12 ( 0.44 ) |
| paraesthesia | 2 | 12.92 ( 3.2 - 52.18 ) | 12.76 ( 21.67 ) | 12.75 ( 3.96 ) | 3.67 ( 1.99 ) |
| malaise | 2 | 3.38 ( 0.84 - 13.66 ) | 3.35 ( 3.31 ) | 3.35 ( 1.04 ) | 1.74 ( 0.06 ) |
| haematotoxicity | 1 | 22.83 ( 3.19 - 163.46 ) | 22.68 ( 20.69 ) | 22.64 ( 4.36 ) | 4.5 ( 2.81 ) |
| infection | 1 | 3.66 ( 0.51 - 26.16 ) | 3.64 ( 1.92 ) | 3.64 ( 0.7 ) | 1.86 ( 0.18 ) |
| toxicity to various agents | 1 | 1.13 ( 0.16 - 8.05 ) | 1.13 ( 0.01 ) | 1.13 ( 0.22 ) | 0.17 ( -1.51 ) |
| dyspnoea exertional | 1 | 55.21 ( 7.69 - 396.43 ) | 54.84 ( 52.61 ) | 54.58 ( 10.49 ) | 5.77 ( 4.08 ) |
| pneumonitis | 1 | 29.75 ( 4.15 - 213.12 ) | 29.55 ( 27.52 ) | 29.48 ( 5.67 ) | 4.88 ( 3.19 ) |
| laryngeal oedema | 1 | 60.97 ( 8.49 - 438.04 ) | 60.57 ( 58.27 ) | 60.24 ( 11.57 ) | 5.91 ( 4.22 ) |
| disease recurrence | 1 | 8.7 ( 1.22 - 62.25 ) | 8.65 ( 6.77 ) | 8.65 ( 1.67 ) | 3.11 ( 1.43 ) |
| hypogammaglobulinaemia | 1 | 20.17 ( 2.82 - 144.4 ) | 20.04 ( 18.07 ) | 20.01 ( 3.85 ) | 4.32 ( 2.64 ) |
| pneumonia | 1 | 1.55 ( 0.22 - 11.05 ) | 1.54 ( 0.19 ) | 1.54 ( 0.3 ) | 0.62 ( -1.06 ) |
| thrombocytopenia | 1 | 3.16 ( 0.44 - 22.62 ) | 3.15 ( 1.47 ) | 3.15 ( 0.61 ) | 1.66 ( -0.03 ) |
| blood immunoglobulin g decreased | 1 | 118.05 ( 16.35 - 852.42 ) | 117.27 ( 114.08 ) | 116.05 ( 22.19 ) | 6.86 ( 5.15 ) |
| aggression | 1 | 1.88 ( 0.26 - 13.43 ) | 1.87 ( 0.41 ) | 1.87 ( 0.36 ) | 0.9 ( -0.78 ) |
| asthma | 1 | 3.88 ( 0.54 - 27.74 ) | 3.86 ( 2.12 ) | 3.86 ( 0.74 ) | 1.95 ( 0.27 ) |
| lip swelling | 1 | 7.63 ( 1.07 - 54.57 ) | 7.59 ( 5.72 ) | 7.58 ( 1.46 ) | 2.92 ( 1.24 ) |
| muscle spasms | 1 | 6.61 ( 0.92 - 47.24 ) | 6.57 ( 4.72 ) | 6.57 ( 1.27 ) | 2.72 ( 1.03 ) |
| oedema | 1 | 10.77 ( 1.51 - 77.02 ) | 10.7 ( 8.79 ) | 10.69 ( 2.06 ) | 3.42 ( 1.73 ) |
| wheezing | 1 | 6.96 ( 0.97 - 49.77 ) | 6.92 ( 5.07 ) | 6.92 ( 1.33 ) | 2.79 ( 1.11 ) |
| heart transplant rejection | 1 | 163.2 ( 22.51 - 1183.02 ) | 162.11 ( 157.81 ) | 159.78 ( 30.46 ) | 7.32 ( 5.6 ) |
| swelling face | 1 | 3.88 ( 0.54 - 27.72 ) | 3.86 ( 2.12 ) | 3.86 ( 0.74 ) | 1.95 ( 0.26 ) |
| erythema | 1 | 0.63 ( 0.09 - 4.5 ) | 0.63 ( 0.22 ) | 0.63 ( 0.12 ) | -0.66 ( -2.35 ) |
| pharyngeal swelling | 1 | 76.53 ( 10.64 - 550.58 ) | 76.03 ( 73.54 ) | 75.51 ( 14.49 ) | 6.24 ( 4.54 ) |
| oropharyngeal discomfort | 1 | 60.97 ( 8.49 - 438.04 ) | 60.57 ( 58.27 ) | 60.24 ( 11.57 ) | 5.91 ( 4.22 ) |
| oxygen saturation decreased | 1 | 4.29 ( 0.6 - 30.64 ) | 4.26 ( 2.5 ) | 4.26 ( 0.82 ) | 2.09 ( 0.41 ) |
| respiratory disorder | 1 | 8.38 ( 1.17 - 59.89 ) | 8.33 ( 6.45 ) | 8.32 ( 1.6 ) | 3.06 ( 1.37 ) |
| back pain | 1 | 4.92 ( 0.69 - 35.15 ) | 4.89 ( 3.1 ) | 4.89 ( 0.94 ) | 2.29 ( 0.61 ) |
| myalgia | 1 | 6.96 ( 0.97 - 49.74 ) | 6.92 ( 5.06 ) | 6.91 ( 1.33 ) | 2.79 ( 1.11 ) |

Abbreviation: Asterisks (*) indicate statistically significant signals in algorithm; ROR, reporting odds ratio; PRR, proportional reporting ratio; EBGM, empirical Bayesian geometric mean; EBGM05, the lower limit of the 95% CI of EBGM; IC, information component; IC025, the lower limit of the 95% CI of the IC; CI, confidence interval; PT, preferred term.

**Supplementary Table 7**: Top 50 most frequent adverse events for Ofatumumab at the PT level in patients aged 18 to 65 from FAERS data

| PT | Case numbers | ROR(95%CI) | PRR(χ^2^) | EBGM(EBGM05) | IC(IC025) |
| --- | --- | --- | --- | --- | --- |
| Fatigue | 2214 | 3.81 ( 3.65 - 3.97 ) | 3.66 ( 4308.04 ) | 3.64 ( 3.51 ) | 1.86 ( 0.2 ) |
| Headache | 1934 | 3.57 ( 3.41 - 3.74 ) | 3.46 ( 3392.49 ) | 3.44 ( 3.31 ) | 1.78 ( 0.11 ) |
| Chills | 1540 | 16 ( 15.2 - 16.85 ) | 15.47 ( 20142.36 ) | 14.95 ( 14.32 ) | 3.9 ( 2.24 ) |
| Pyrexia | 1486 | 5.41 ( 5.13 - 5.69 ) | 5.25 ( 5088.4 ) | 5.2 ( 4.98 ) | 2.38 ( 0.71 ) |
| Pain | 1474 | 3.21 ( 3.05 - 3.39 ) | 3.14 ( 2153.92 ) | 3.12 ( 2.99 ) | 1.64 ( -0.02 ) |
| Influenza like illness | 1187 | 15.61 ( 14.72 - 16.55 ) | 15.21 ( 15227.41 ) | 14.71 ( 14 ) | 3.88 ( 2.21 ) |
| Nausea | 730 | 1.14 ( 1.06 - 1.23 ) | 1.14 ( 12.89 ) | 1.14 ( 1.07 ) | 0.19 ( -1.48 ) |
| Covid-19 | 635 | 4.31 ( 3.98 - 4.66 ) | 4.26 ( 1574.47 ) | 4.23 ( 3.96 ) | 2.08 ( 0.41 ) |
| Hypoaesthesia | 572 | 4.07 ( 3.75 - 4.43 ) | 4.03 ( 1297.01 ) | 4 ( 3.74 ) | 2 ( 0.34 ) |
| Asthenia | 567 | 2.29 ( 2.11 - 2.49 ) | 2.27 ( 403.91 ) | 2.27 ( 2.11 ) | 1.18 ( -0.49 ) |
| Dizziness | 504 | 1.4 ( 1.28 - 1.53 ) | 1.39 ( 56.46 ) | 1.39 ( 1.29 ) | 0.48 ( -1.19 ) |
| Gait disturbance | 489 | 3.46 ( 3.16 - 3.79 ) | 3.43 ( 839.11 ) | 3.41 ( 3.17 ) | 1.77 ( 0.11 ) |
| Multiple sclerosis relapse | 477 | 5.63 ( 5.14 - 6.16 ) | 5.58 ( 1771.22 ) | 5.52 ( 5.11 ) | 2.46 ( 0.8 ) |
| Feeling abnormal | 475 | 2.62 ( 2.39 - 2.87 ) | 2.6 ( 467.35 ) | 2.59 ( 2.4 ) | 1.37 ( -0.29 ) |
| Pain in extremity | 471 | 1.96 ( 1.79 - 2.15 ) | 1.95 ( 217.68 ) | 1.94 ( 1.8 ) | 0.96 ( -0.71 ) |
| Nasopharyngitis | 447 | 2.83 ( 2.58 - 3.11 ) | 2.81 ( 520.86 ) | 2.8 ( 2.59 ) | 1.49 ( -0.18 ) |
| Malaise | 434 | 1.37 ( 1.24 - 1.5 ) | 1.36 ( 42.31 ) | 1.36 ( 1.26 ) | 0.45 ( -1.22 ) |
| Injection site pain | 427 | 1.7 ( 1.54 - 1.87 ) | 1.69 ( 120.36 ) | 1.69 ( 1.56 ) | 0.75 ( -0.91 ) |
| Arthralgia | 390 | 1.14 ( 1.03 - 1.26 ) | 1.14 ( 6.71 ) | 1.14 ( 1.05 ) | 0.19 ( -1.48 ) |
| Myalgia | 390 | 3.05 ( 2.76 - 3.37 ) | 3.03 ( 528.56 ) | 3.02 ( 2.77 ) | 1.59 ( -0.07 ) |
| Back pain | 376 | 2.08 ( 1.87 - 2.3 ) | 2.07 ( 206.65 ) | 2.06 ( 1.89 ) | 1.04 ( -0.62 ) |
| Cough | 368 | 1.87 ( 1.69 - 2.07 ) | 1.86 ( 147.07 ) | 1.86 ( 1.71 ) | 0.89 ( -0.77 ) |
| Inappropriate schedule of product administration | 357 | 3.26 ( 2.94 - 3.62 ) | 3.24 ( 551.47 ) | 3.23 ( 2.96 ) | 1.69 ( 0.02 ) |
| Vomiting | 356 | 0.98 ( 0.88 - 1.09 ) | 0.98 ( 0.17 ) | 0.98 ( 0.9 ) | -0.03 ( -1.7 ) |
| Muscle spasms | 330 | 2.33 ( 2.09 - 2.6 ) | 2.32 ( 247.38 ) | 2.31 ( 2.11 ) | 1.21 ( -0.46 ) |
| Muscular weakness | 324 | 3.68 ( 3.3 - 4.11 ) | 3.66 ( 622.28 ) | 3.64 ( 3.32 ) | 1.86 ( 0.2 ) |
| Paraesthesia | 321 | 2.08 ( 1.87 - 2.32 ) | 2.07 ( 178.37 ) | 2.07 ( 1.89 ) | 1.05 ( -0.62 ) |
| Fall | 318 | 1.82 ( 1.63 - 2.04 ) | 1.82 ( 116.75 ) | 1.81 ( 1.65 ) | 0.86 ( -0.81 ) |
| Urinary tract infection | 316 | 2.78 ( 2.49 - 3.11 ) | 2.77 ( 355.47 ) | 2.76 ( 2.51 ) | 1.46 ( -0.2 ) |
| Accidental exposure to product | 296 | 4.89 ( 4.36 - 5.48 ) | 4.86 ( 898.37 ) | 4.82 ( 4.37 ) | 2.27 ( 0.6 ) |
| Tremor | 294 | 2.46 ( 2.19 - 2.76 ) | 2.45 ( 250.93 ) | 2.44 ( 2.22 ) | 1.29 ( -0.38 ) |
| Balance disorder | 264 | 4.34 ( 3.85 - 4.91 ) | 4.32 ( 668.36 ) | 4.29 ( 3.87 ) | 2.1 ( 0.43 ) |
| Oropharyngeal pain | 252 | 2.86 ( 2.53 - 3.24 ) | 2.85 ( 301.7 ) | 2.84 ( 2.56 ) | 1.51 ( -0.16 ) |
| Drug ineffective | 251 | 0.32 ( 0.28 - 0.37 ) | 0.33 ( 354.71 ) | 0.33 ( 0.29 ) | -1.61 ( -3.28 ) |
| Injection site bruising | 248 | 3.55 ( 3.13 - 4.02 ) | 3.53 ( 447.09 ) | 3.51 ( 3.16 ) | 1.81 ( 0.15 ) |
| Insomnia | 239 | 1.13 ( 1 - 1.28 ) | 1.13 ( 3.58 ) | 1.13 ( 1.02 ) | 0.18 ( -1.49 ) |
| Product dose omission issue | 238 | 1.55 ( 1.36 - 1.76 ) | 1.54 ( 45.57 ) | 1.54 ( 1.39 ) | 0.62 ( -1.04 ) |
| Migraine | 229 | 2.48 ( 2.18 - 2.83 ) | 2.48 ( 200.83 ) | 2.47 ( 2.21 ) | 1.3 ( -0.36 ) |
| Diarrhoea | 222 | 0.51 ( 0.44 - 0.58 ) | 0.51 ( 106.6 ) | 0.51 ( 0.46 ) | -0.97 ( -2.64 ) |
| Pruritus | 220 | 0.81 ( 0.71 - 0.92 ) | 0.81 ( 10.26 ) | 0.81 ( 0.72 ) | -0.31 ( -1.98 ) |
| Dyspnoea | 215 | 0.54 ( 0.47 - 0.62 ) | 0.54 ( 84.3 ) | 0.54 ( 0.48 ) | -0.88 ( -2.55 ) |
| Illness | 211 | 4.35 ( 3.8 - 4.98 ) | 4.33 ( 535.87 ) | 4.3 ( 3.84 ) | 2.1 ( 0.44 ) |
| Memory impairment | 203 | 2.01 ( 1.75 - 2.31 ) | 2.01 ( 102.13 ) | 2 ( 1.78 ) | 1 ( -0.67 ) |
| Rash | 199 | 0.67 ( 0.58 - 0.77 ) | 0.67 ( 31.92 ) | 0.67 ( 0.6 ) | -0.57 ( -2.24 ) |
| Rhinorrhoea | 192 | 4 ( 3.47 - 4.62 ) | 3.99 ( 426.82 ) | 3.96 ( 3.52 ) | 1.99 ( 0.32 ) |
| Limb discomfort | 184 | 7.28 ( 6.29 - 8.43 ) | 7.26 ( 976.13 ) | 7.15 ( 6.33 ) | 2.84 ( 1.17 ) |
| Device malfunction | 174 | 4.55 ( 3.91 - 5.28 ) | 4.53 ( 474.33 ) | 4.49 ( 3.97 ) | 2.17 ( 0.5 ) |
| Influenza | 172 | 1.9 ( 1.64 - 2.21 ) | 1.9 ( 72.69 ) | 1.89 ( 1.67 ) | 0.92 ( -0.75 ) |
| Injection site haemorrhage | 167 | 2.49 ( 2.14 - 2.9 ) | 2.49 ( 147.98 ) | 2.48 ( 2.18 ) | 1.31 ( -0.36 ) |
| Stress | 163 | 2.52 ( 2.16 - 2.94 ) | 2.51 ( 147.54 ) | 2.5 ( 2.2 ) | 1.32 ( -0.34 ) |

Abbreviation: Asterisks (*) indicate statistically significant signals in algorithm; ROR, reporting odds ratio; PRR, proportional reporting ratio; EBGM, empirical Bayesian geometric mean; EBGM05, the lower limit of the 95% CI of EBGM; IC, information component; IC025, the lower limit of the 95% CI of the IC; CI, confidence interval; PT, preferred term.

**Supplementary Table 8**: Top 50 most frequent adverse events for Ofatumumab at the PT level in patients aged 65 to 85 from FAERS data

| PT | Case numbers | ROR(95%CI) | PRR(χ^2^) | EBGM(EBGM05) | IC(IC025) |
| --- | --- | --- | --- | --- | --- |
| Pyrexia | 149 | 5.96 ( 5.06 - 7.02 ) | 5.79 ( 592.38 ) | 5.78 ( 5.04 ) | 2.53 ( 0.86 ) |
| Pneumonia | 120 | 3.42 ( 2.85 - 4.1 ) | 3.35 ( 199.25 ) | 3.35 ( 2.88 ) | 1.74 ( 0.08 ) |
| Febrile neutropenia | 100 | 16.34 ( 13.4 - 19.94 ) | 15.99 ( 1397.49 ) | 15.89 ( 13.45 ) | 3.99 ( 2.32 ) |
| Fatigue | 97 | 1.67 ( 1.36 - 2.04 ) | 1.65 ( 25.28 ) | 1.65 ( 1.4 ) | 0.72 ( -0.94 ) |
| Headache | 91 | 2.66 ( 2.16 - 3.27 ) | 2.62 ( 91.93 ) | 2.62 ( 2.2 ) | 1.39 ( -0.28 ) |
| Chills | 90 | 10 ( 8.11 - 12.32 ) | 9.81 ( 710.57 ) | 9.77 ( 8.2 ) | 3.29 ( 1.62 ) |
| Pain | 59 | 1.68 ( 1.3 - 2.17 ) | 1.67 ( 15.98 ) | 1.67 ( 1.35 ) | 0.74 ( -0.93 ) |
| Nausea | 55 | 1.03 ( 0.79 - 1.34 ) | 1.03 ( 0.05 ) | 1.03 ( 0.82 ) | 0.04 ( -1.62 ) |
| Dizziness | 53 | 1.3 ( 0.99 - 1.7 ) | 1.29 ( 3.57 ) | 1.29 ( 1.03 ) | 0.37 ( -1.29 ) |
| Asthenia | 52 | 1.36 ( 1.04 - 1.79 ) | 1.36 ( 4.96 ) | 1.36 ( 1.08 ) | 0.44 ( -1.22 ) |
| Dyspnoea | 52 | 0.96 ( 0.73 - 1.26 ) | 0.96 ( 0.09 ) | 0.96 ( 0.76 ) | -0.06 ( -1.73 ) |
| Death | 45 | 0.53 ( 0.4 - 0.71 ) | 0.54 ( 18.4 ) | 0.54 ( 0.42 ) | -0.9 ( -2.57 ) |
| Malaise | 45 | 1.26 ( 0.94 - 1.69 ) | 1.25 ( 2.33 ) | 1.25 ( 0.98 ) | 0.33 ( -1.34 ) |
| Gait disturbance | 45 | 2.56 ( 1.91 - 3.44 ) | 2.55 ( 42.34 ) | 2.54 ( 1.99 ) | 1.35 ( -0.32 ) |
| Influenza like illness | 43 | 11.18 ( 8.27 - 15.11 ) | 11.08 ( 392.58 ) | 11.03 ( 8.57 ) | 3.46 ( 1.8 ) |
| Covid-19 | 43 | 2.8 ( 2.07 - 3.78 ) | 2.78 ( 49.1 ) | 2.78 ( 2.16 ) | 1.47 ( -0.19 ) |
| Fall | 40 | 0.95 ( 0.7 - 1.3 ) | 0.95 ( 0.09 ) | 0.95 ( 0.73 ) | -0.07 ( -1.73 ) |
| Cough | 35 | 1.43 ( 1.03 - 2 ) | 1.43 ( 4.53 ) | 1.43 ( 1.08 ) | 0.51 ( -1.15 ) |
| Urinary tract infection | 35 | 1.97 ( 1.41 - 2.74 ) | 1.96 ( 16.5 ) | 1.96 ( 1.48 ) | 0.97 ( -0.7 ) |
| Diarrhoea | 34 | 0.56 ( 0.4 - 0.79 ) | 0.56 ( 11.55 ) | 0.57 ( 0.43 ) | -0.82 ( -2.49 ) |
| Multiple sclerosis relapse | 34 | 31.5 ( 22.43 - 44.25 ) | 31.27 ( 982.38 ) | 30.84 ( 23.21 ) | 4.95 ( 3.28 ) |
| Neutropenia | 33 | 2.69 ( 1.91 - 3.79 ) | 2.68 ( 34.71 ) | 2.67 ( 2.01 ) | 1.42 ( -0.25 ) |
| Infusion related reaction | 33 | 9.49 ( 6.73 - 13.38 ) | 9.43 ( 247.7 ) | 9.39 ( 7.05 ) | 3.23 ( 1.56 ) |
| Vomiting | 33 | 1.06 ( 0.75 - 1.49 ) | 1.05 ( 0.09 ) | 1.05 ( 0.79 ) | 0.08 ( -1.59 ) |
| Pain in extremity | 33 | 1.28 ( 0.91 - 1.81 ) | 1.28 ( 2.05 ) | 1.28 ( 0.96 ) | 0.36 ( -1.31 ) |
| Feeling abnormal | 32 | 2.01 ( 1.42 - 2.85 ) | 2 ( 16.09 ) | 2 ( 1.5 ) | 1 ( -0.67 ) |
| Balance disorder | 32 | 3.82 ( 2.7 - 5.41 ) | 3.8 ( 66.01 ) | 3.79 ( 2.84 ) | 1.92 ( 0.26 ) |
| Sepsis | 29 | 2.52 ( 1.75 - 3.63 ) | 2.51 ( 26.38 ) | 2.51 ( 1.85 ) | 1.33 ( -0.34 ) |
| Neutropenic sepsis | 29 | 33.89 ( 23.46 - 48.97 ) | 33.67 ( 905.69 ) | 33.18 ( 24.39 ) | 5.05 ( 3.38 ) |
| Anaemia | 28 | 1.24 ( 0.86 - 1.8 ) | 1.24 ( 1.33 ) | 1.24 ( 0.91 ) | 0.31 ( -1.35 ) |
| Back pain | 28 | 1.42 ( 0.98 - 2.06 ) | 1.42 ( 3.47 ) | 1.42 ( 1.04 ) | 0.5 ( -1.16 ) |
| Tremor | 26 | 2.05 ( 1.39 - 3.01 ) | 2.04 ( 13.87 ) | 2.04 ( 1.48 ) | 1.03 ( -0.64 ) |
| Muscular weakness | 25 | 2.46 ( 1.66 - 3.65 ) | 2.45 ( 21.53 ) | 2.45 ( 1.76 ) | 1.29 ( -0.37 ) |
| Infection | 25 | 2.53 ( 1.71 - 3.75 ) | 2.52 ( 22.93 ) | 2.52 ( 1.81 ) | 1.33 ( -0.33 ) |
| Hypotension | 23 | 1.12 ( 0.74 - 1.68 ) | 1.11 ( 0.27 ) | 1.11 ( 0.79 ) | 0.16 ( -1.51 ) |
| Rash | 23 | 0.82 ( 0.54 - 1.23 ) | 0.82 ( 0.92 ) | 0.82 ( 0.58 ) | -0.29 ( -1.95 ) |
| Dehydration | 22 | 1.54 ( 1.02 - 2.35 ) | 1.54 ( 4.19 ) | 1.54 ( 1.08 ) | 0.62 ( -1.04 ) |
| Drug ineffective | 21 | 0.33 ( 0.21 - 0.5 ) | 0.33 ( 28.91 ) | 0.33 ( 0.23 ) | -1.6 ( -3.26 ) |
| Paraesthesia | 21 | 2.48 ( 1.62 - 3.81 ) | 2.48 ( 18.5 ) | 2.47 ( 1.73 ) | 1.31 ( -0.36 ) |
| Chest pain | 20 | 1.5 ( 0.96 - 2.32 ) | 1.49 ( 3.26 ) | 1.49 ( 1.03 ) | 0.58 ( -1.09 ) |
| Inappropriate schedule of product administration | 20 | 2.11 ( 1.36 - 3.28 ) | 2.11 ( 11.62 ) | 2.1 ( 1.46 ) | 1.07 ( -0.59 ) |
| Urticaria | 19 | 2.32 ( 1.48 - 3.64 ) | 2.31 ( 14.17 ) | 2.31 ( 1.58 ) | 1.21 ( -0.46 ) |
| White blood cell count decreased | 19 | 1.75 ( 1.12 - 2.75 ) | 1.75 ( 6.13 ) | 1.75 ( 1.2 ) | 0.81 ( -0.86 ) |
| Hypoaesthesia | 19 | 2.24 ( 1.43 - 3.52 ) | 2.24 ( 13.04 ) | 2.24 ( 1.53 ) | 1.16 ( -0.5 ) |
| Hypertension | 18 | 1 ( 0.63 - 1.58 ) | 1 ( 0 ) | 1 ( 0.68 ) | -0.01 ( -1.67 ) |
| Hypersensitivity | 18 | 1.98 ( 1.25 - 3.15 ) | 1.98 ( 8.71 ) | 1.98 ( 1.34 ) | 0.98 ( -0.68 ) |
| Muscle spasms | 18 | 1.26 ( 0.79 - 2 ) | 1.26 ( 0.95 ) | 1.26 ( 0.85 ) | 0.33 ( -1.34 ) |
| Progressive multifocal leukoencephalopathy | 17 | 26 ( 16.1 - 41.97 ) | 25.9 ( 402.26 ) | 25.61 ( 17.15 ) | 4.68 ( 3.01 ) |
| Neutrophil count decreased | 17 | 3.83 ( 2.38 - 6.17 ) | 3.82 ( 35.34 ) | 3.81 ( 2.56 ) | 1.93 ( 0.26 ) |
| Lower respiratory tract infection | 17 | 5.44 ( 3.38 - 8.77 ) | 5.42 ( 61.25 ) | 5.41 ( 3.63 ) | 2.44 ( 0.77 ) |

Abbreviation: Asterisks (*) indicate statistically significant signals in algorithm; ROR, reporting odds ratio; PRR, proportional reporting ratio; EBGM, empirical Bayesian geometric mean; EBGM05, the lower limit of the 95% CI of EBGM; IC, information component; IC025, the lower limit of the 95% CI of the IC; CI, confidence interval; PT, preferred term.

. **Supplementary Table 9**: Top 50 most frequent adverse events for Ofatumumab at the PT level in patients aged over 85 from FAERS data

| PT | Case numbers | ROR(95%CI) | PRR(χ^2^) | EBGM(EBGM05) | IC(IC025) |
| --- | --- | --- | --- | --- | --- |
| Pyrexia | 5 | 10.98 ( 4.49 - 26.89 ) | 10.58 ( 43.47 ) | 10.56 ( 4.99 ) | 3.4 ( 1.71 ) |
| Malignant neoplasm progression | 5 | 29.93 ( 12.21 - 73.37 ) | 28.76 ( 133.6 ) | 28.64 ( 13.53 ) | 4.84 ( 3.15 ) |
| Pneumonia | 4 | 3.54 ( 1.31 - 9.6 ) | 3.46 ( 7.06 ) | 3.46 ( 1.5 ) | 1.79 ( 0.1 ) |
| Febrile neutropenia | 4 | 84.53 ( 31.03 - 230.29 ) | 81.84 ( 315.57 ) | 80.84 ( 34.95 ) | 6.34 ( 4.64 ) |
| Infusion related reaction | 3 | 57.16 ( 18.09 - 180.56 ) | 55.8 ( 160.15 ) | 55.33 ( 21.13 ) | 5.79 ( 4.1 ) |
| Oxygen saturation decreased | 3 | 17.91 ( 5.69 - 56.41 ) | 17.5 ( 46.63 ) | 17.46 ( 6.69 ) | 4.13 ( 2.44 ) |
| Chronic lymphocytic leukaemia | 3 | 295.97 ( 91.86 - 953.56 ) | 288.83 ( 824.22 ) | 276.67 ( 103.95 ) | 8.11 ( 6.39 ) |
| Rash | 3 | 4.83 ( 1.54 - 15.2 ) | 4.74 ( 8.89 ) | 4.74 ( 1.82 ) | 2.24 ( 0.56 ) |
| Asthma | 2 | 18.31 ( 4.52 - 74.21 ) | 18.04 ( 32.12 ) | 17.99 ( 5.58 ) | 4.17 ( 2.48 ) |
| Dyspnoea | 2 | 1.39 ( 0.34 - 5.63 ) | 1.39 ( 0.22 ) | 1.39 ( 0.43 ) | 0.47 ( -1.22 ) |
| Lymphocyte count increased | 2 | 633.72 ( 146.99 - 2732.14 ) | 623.51 ( 1134.97 ) | 569.38 ( 167.65 ) | 9.15 ( 7.37 ) |
| Therapeutic response decreased | 2 | 32.21 ( 7.94 - 130.68 ) | 31.7 ( 59.21 ) | 31.56 ( 9.78 ) | 4.98 ( 3.29 ) |
| Atrioventricular block complete | 2 | 32.21 ( 7.94 - 130.68 ) | 31.7 ( 59.21 ) | 31.56 ( 9.78 ) | 4.98 ( 3.29 ) |
| Death | 2 | 0.32 ( 0.08 - 1.29 ) | 0.33 ( 2.84 ) | 0.33 ( 0.1 ) | -1.59 ( -3.28 ) |
| Pelvic infection | 2 | 6654.16 ( 929.81 - 47620.36 ) | 6546.85 ( 6544.89 ) | 3273.93 ( 630.83 ) | 11.68 ( 9.55 ) |
| Abdominal pain | 2 | 6.29 ( 1.55 - 25.45 ) | 6.2 ( 8.74 ) | 6.2 ( 1.92 ) | 2.63 ( 0.94 ) |
| Chills | 2 | 13.28 ( 3.28 - 53.77 ) | 13.08 ( 22.3 ) | 13.06 ( 4.05 ) | 3.71 ( 2.02 ) |
| Renal failure | 2 | 4.68 ( 1.16 - 18.95 ) | 4.62 ( 5.7 ) | 4.62 ( 1.43 ) | 2.21 ( 0.52 ) |
| Tumour lysis syndrome | 2 | 146.23 ( 35.62 - 600.31 ) | 143.89 ( 277.72 ) | 140.81 ( 43.2 ) | 7.14 ( 5.43 ) |
| Haematochezia | 1 | 5.36 ( 0.75 - 38.4 ) | 5.33 ( 3.52 ) | 5.32 ( 1.03 ) | 2.41 ( 0.72 ) |
| Gastrointestinal necrosis | 1 | 206.24 ( 27.96 - 1521.24 ) | 204.59 ( 196.46 ) | 198.42 ( 37.28 ) | 7.63 ( 5.88 ) |
| Influenza like illness | 1 | 27.04 ( 3.76 - 194.28 ) | 26.83 ( 24.77 ) | 26.73 ( 5.13 ) | 4.74 ( 3.04 ) |
| Gastrointestinal angiodysplasia | 1 | 286.95 ( 38.45 - 2141.49 ) | 284.65 ( 270.88 ) | 272.83 ( 50.76 ) | 8.09 ( 6.31 ) |
| Feeling hot | 1 | 17.31 ( 2.41 - 124.22 ) | 17.18 ( 15.21 ) | 17.14 ( 3.3 ) | 4.1 ( 2.41 ) |
| Wheezing | 1 | 8.03 ( 1.12 - 57.54 ) | 7.97 ( 6.1 ) | 7.97 ( 1.53 ) | 2.99 ( 1.31 ) |
| Brain oedema | 1 | 70.96 ( 9.81 - 513.14 ) | 70.4 ( 67.69 ) | 69.66 ( 13.31 ) | 6.12 ( 4.41 ) |
| Confusional state | 1 | 1.01 ( 0.14 - 7.25 ) | 1.01 ( 0 ) | 1.01 ( 0.2 ) | 0.02 ( -1.67 ) |
| Meningioma | 1 | 178.37 ( 24.28 - 1310.33 ) | 176.94 ( 170.35 ) | 172.31 ( 32.48 ) | 7.43 ( 5.68 ) |
| Atrial fibrillation | 1 | 1.95 ( 0.27 - 13.97 ) | 1.94 ( 0.46 ) | 1.94 ( 0.37 ) | 0.96 ( -0.73 ) |
| Adverse event | 1 | 6.69 ( 0.93 - 47.94 ) | 6.65 ( 4.8 ) | 6.64 ( 1.28 ) | 2.73 ( 1.04 ) |
| Sudden death | 1 | 23.82 ( 3.32 - 171.05 ) | 23.63 ( 21.61 ) | 23.55 ( 4.53 ) | 4.56 ( 2.86 ) |
| Gastrointestinal infection | 1 | 72.52 ( 10.03 - 524.54 ) | 71.94 ( 69.2 ) | 71.17 ( 13.59 ) | 6.15 ( 4.44 ) |
| Condition aggravated | 1 | 2.03 ( 0.28 - 14.52 ) | 2.02 ( 0.52 ) | 2.02 ( 0.39 ) | 1.01 ( -0.67 ) |
| General physical health deterioration | 1 | 2.07 ( 0.29 - 14.8 ) | 2.06 ( 0.55 ) | 2.06 ( 0.4 ) | 1.04 ( -0.64 ) |
| Pulmonary mass | 1 | 43.41 ( 6.03 - 312.67 ) | 43.07 ( 40.83 ) | 42.8 ( 8.2 ) | 5.42 ( 3.72 ) |
| Metastatic neoplasm | 1 | 89.18 ( 12.3 - 646.63 ) | 88.47 ( 85.34 ) | 87.3 ( 16.64 ) | 6.45 ( 4.73 ) |
| Hepatocellular carcinoma | 1 | 143.47 ( 19.63 - 1048.58 ) | 142.32 ( 137.35 ) | 139.32 ( 26.38 ) | 7.12 ( 5.39 ) |
| Second primary malignancy | 1 | 95.65 ( 13.18 - 694.15 ) | 94.88 ( 91.57 ) | 93.54 ( 17.81 ) | 6.55 ( 4.83 ) |
| Abdominal distension | 1 | 7.12 ( 0.99 - 51 ) | 7.07 ( 5.21 ) | 7.06 ( 1.36 ) | 2.82 ( 1.13 ) |
| Decreased appetite | 1 | 1.31 ( 0.18 - 9.39 ) | 1.31 ( 0.07 ) | 1.31 ( 0.25 ) | 0.39 ( -1.3 ) |
| Haematotoxicity | 1 | 164.99 ( 22.5 - 1209.68 ) | 163.67 ( 157.74 ) | 159.7 ( 30.16 ) | 7.32 ( 5.58 ) |
| Infection | 1 | 5.21 ( 0.73 - 37.28 ) | 5.17 ( 3.37 ) | 5.17 ( 1 ) | 2.37 ( 0.68 ) |
| Large intestinal obstruction | 1 | 212.9 ( 28.84 - 1571.84 ) | 211.19 ( 202.66 ) | 204.62 ( 38.41 ) | 7.68 ( 5.92 ) |
| Nausea | 1 | 0.97 ( 0.14 - 6.98 ) | 0.97 ( 0 ) | 0.97 ( 0.19 ) | -0.04 ( -1.72 ) |
| Neutrophil count decreased | 1 | 21.35 ( 2.97 - 153.27 ) | 21.19 ( 19.18 ) | 21.12 ( 4.06 ) | 4.4 ( 2.71 ) |
| Oedema mucosal | 1 | 1320.01 ( 153.1 - 11381.31 ) | 1309.37 ( 1089.48 ) | 1091.31 ( 179.92 ) | 10.09 ( 8.08 ) |
| Squamous cell carcinoma of skin | 1 | 63.45 ( 8.78 - 458.35 ) | 62.95 ( 60.39 ) | 62.36 ( 11.92 ) | 5.96 ( 4.25 ) |
| Cholangiocarcinoma | 1 | 550 ( 70.97 - 4262.41 ) | 545.57 ( 501.77 ) | 503.68 ( 90.79 ) | 8.98 ( 7.12 ) |
| Hepatic cancer | 1 | 29.07 ( 4.04 - 208.9 ) | 28.84 ( 26.77 ) | 28.72 ( 5.51 ) | 4.84 ( 3.15 ) |
| Neoplasm malignant | 1 | 9.25 ( 1.29 - 66.27 ) | 9.18 ( 7.29 ) | 9.17 ( 1.77 ) | 3.2 ( 1.51 ) |

Abbreviation: Asterisks (*) indicate statistically significant signals in algorithm; ROR, reporting odds ratio; PRR, proportional reporting ratio; EBGM, empirical Bayesian geometric mean; EBGM05, the lower limit of the 95% CI of EBGM; IC, information component; IC025, the lower limit of the 95% CI of the IC; CI, confidence interval; PT, preferred term.

**Supplementary Table 10**: Top 50 most frequent adverse events for Ofatumumab at the PT level in patients reported by consumers from FAERS data

| PT | Case numbers | ROR(95%CI) | PRR(χ^2^) | EBGM(EBGM05) | IC(IC025) |
| --- | --- | --- | --- | --- | --- |
| Fatigue | 4235 | 3.61 ( 3.5 - 3.72 ) | 3.46 ( 7445.5 ) | 3.43 ( 3.34 ) | 1.78 ( 0.11 ) |
| Headache | 3685 | 3.95 ( 3.82 - 4.08 ) | 3.81 ( 7620.86 ) | 3.77 ( 3.67 ) | 1.91 ( 0.25 ) |
| Chills | 2873 | 21.23 ( 20.42 - 22.06 ) | 20.47 ( 49740.51 ) | 19.17 ( 18.56 ) | 4.26 ( 2.59 ) |
| Pain | 2772 | 3.19 ( 3.07 - 3.31 ) | 3.11 ( 3964.96 ) | 3.08 ( 2.99 ) | 1.62 ( -0.04 ) |
| Pyrexia | 2547 | 7.81 ( 7.51 - 8.13 ) | 7.58 ( 14248.36 ) | 7.41 ( 7.17 ) | 2.89 ( 1.22 ) |
| Influenza like illness | 2196 | 17.25 ( 16.51 - 18.02 ) | 16.78 ( 30843.48 ) | 15.91 ( 15.34 ) | 3.99 ( 2.33 ) |
| Nausea | 1299 | 1.21 ( 1.14 - 1.28 ) | 1.2 ( 45.66 ) | 1.2 ( 1.15 ) | 0.27 ( -1.4 ) |
| Covid-19 | 1086 | 3.93 ( 3.7 - 4.18 ) | 3.89 ( 2310.64 ) | 3.85 ( 3.66 ) | 1.95 ( 0.28 ) |
| Asthenia | 1040 | 1.97 ( 1.85 - 2.1 ) | 1.96 ( 487.59 ) | 1.95 ( 1.85 ) | 0.96 ( -0.7 ) |
| Feeling abnormal | 974 | 2.1 ( 1.97 - 2.23 ) | 2.08 ( 546.97 ) | 2.07 ( 1.97 ) | 1.05 ( -0.61 ) |
| Hypoaesthesia | 971 | 4.29 ( 4.03 - 4.58 ) | 4.25 ( 2387.16 ) | 4.2 ( 3.99 ) | 2.07 ( 0.41 ) |
| Gait disturbance | 968 | 2.87 ( 2.69 - 3.06 ) | 2.85 ( 1152.98 ) | 2.83 ( 2.68 ) | 1.5 ( -0.17 ) |
| Dizziness | 881 | 1.17 ( 1.09 - 1.25 ) | 1.17 ( 21.09 ) | 1.17 ( 1.1 ) | 0.22 ( -1.44 ) |
| Multiple sclerosis relapse | 881 | 6.55 ( 6.12 - 7 ) | 6.48 ( 4003.6 ) | 6.36 ( 6.02 ) | 2.67 ( 1 ) |
| Malaise | 867 | 1.16 ( 1.09 - 1.24 ) | 1.16 ( 19.41 ) | 1.16 ( 1.1 ) | 0.21 ( -1.45 ) |
| Nasopharyngitis | 829 | 2.81 ( 2.62 - 3.01 ) | 2.79 ( 944.7 ) | 2.77 ( 2.62 ) | 1.47 ( -0.2 ) |
| Pain in extremity | 822 | 1.71 ( 1.6 - 1.84 ) | 1.71 ( 240.92 ) | 1.7 ( 1.61 ) | 0.77 ( -0.9 ) |
| Injection site pain | 798 | 1.61 ( 1.5 - 1.72 ) | 1.6 ( 180.51 ) | 1.6 ( 1.51 ) | 0.68 ( -0.99 ) |
| Drug ineffective | 717 | 0.31 ( 0.29 - 0.33 ) | 0.32 ( 1098.62 ) | 0.32 ( 0.3 ) | -1.66 ( -3.33 ) |
| Arthralgia | 677 | 1.14 ( 1.06 - 1.23 ) | 1.14 ( 11.74 ) | 1.14 ( 1.07 ) | 0.19 ( -1.48 ) |
| Myalgia | 662 | 2.99 ( 2.77 - 3.23 ) | 2.97 ( 860.93 ) | 2.95 ( 2.77 ) | 1.56 ( -0.1 ) |
| Back pain | 651 | 1.85 ( 1.71 - 2 ) | 1.84 ( 249.68 ) | 1.84 ( 1.72 ) | 0.88 ( -0.79 ) |
| Inappropriate schedule of product administration | 640 | 2.34 ( 2.16 - 2.53 ) | 2.33 ( 482.38 ) | 2.32 ( 2.17 ) | 1.21 ( -0.45 ) |
| Fall | 616 | 1.28 ( 1.18 - 1.38 ) | 1.27 ( 36.09 ) | 1.27 ( 1.19 ) | 0.35 ( -1.32 ) |
| Cough | 603 | 1.44 ( 1.33 - 1.56 ) | 1.44 ( 79.85 ) | 1.43 ( 1.34 ) | 0.52 ( -1.15 ) |
| Muscle spasms | 584 | 1.97 ( 1.82 - 2.14 ) | 1.96 ( 275.38 ) | 1.96 ( 1.83 ) | 0.97 ( -0.7 ) |
| Accidental exposure to product | 583 | 4.34 ( 3.99 - 4.71 ) | 4.31 ( 1462.44 ) | 4.26 ( 3.98 ) | 2.09 ( 0.43 ) |
| Vomiting | 565 | 1 ( 0.92 - 1.08 ) | 1 ( 0 ) | 1 ( 0.93 ) | 0 ( -1.67 ) |
| Paraesthesia | 565 | 2.64 ( 2.43 - 2.87 ) | 2.63 ( 565.37 ) | 2.61 ( 2.44 ) | 1.38 ( -0.28 ) |
| Muscular weakness | 563 | 3.41 ( 3.14 - 3.71 ) | 3.4 ( 942.85 ) | 3.37 ( 3.14 ) | 1.75 ( 0.09 ) |
| Product dose omission issue | 562 | 1.38 ( 1.27 - 1.5 ) | 1.38 ( 59.37 ) | 1.38 ( 1.29 ) | 0.46 ( -1.2 ) |
| Tremor | 542 | 2.26 ( 2.08 - 2.46 ) | 2.25 ( 376.46 ) | 2.24 ( 2.09 ) | 1.17 ( -0.5 ) |
| Illness | 525 | 3.5 ( 3.21 - 3.82 ) | 3.49 ( 921.56 ) | 3.46 ( 3.22 ) | 1.79 ( 0.12 ) |
| Urinary tract infection | 507 | 2.27 ( 2.08 - 2.48 ) | 2.26 ( 354.84 ) | 2.25 ( 2.09 ) | 1.17 ( -0.5 ) |
| Balance disorder | 507 | 3.39 ( 3.1 - 3.7 ) | 3.37 ( 838.52 ) | 3.35 ( 3.11 ) | 1.74 ( 0.08 ) |
| Memory impairment | 475 | 1.77 ( 1.62 - 1.94 ) | 1.76 ( 156.52 ) | 1.76 ( 1.63 ) | 0.81 ( -0.85 ) |
| Oropharyngeal pain | 450 | 2.95 ( 2.69 - 3.24 ) | 2.94 ( 570.07 ) | 2.92 ( 2.7 ) | 1.54 ( -0.12 ) |
| Injection site bruising | 431 | 3.21 ( 2.91 - 3.53 ) | 3.19 ( 643.2 ) | 3.17 ( 2.93 ) | 1.66 ( 0 ) |
| Insomnia | 425 | 0.94 ( 0.85 - 1.03 ) | 0.94 ( 1.64 ) | 0.94 ( 0.87 ) | -0.09 ( -1.76 ) |
| Migraine | 413 | 2.62 ( 2.38 - 2.89 ) | 2.61 ( 408.59 ) | 2.6 ( 2.4 ) | 1.38 ( -0.29 ) |
| Rhinorrhoea | 362 | 3.57 ( 3.21 - 3.96 ) | 3.55 ( 657.24 ) | 3.52 ( 3.23 ) | 1.82 ( 0.15 ) |
| Diarrhoea | 355 | 0.41 ( 0.37 - 0.46 ) | 0.42 ( 295.52 ) | 0.42 ( 0.38 ) | -1.27 ( -2.93 ) |
| Pruritus | 354 | 0.71 ( 0.64 - 0.79 ) | 0.71 ( 40.62 ) | 0.72 ( 0.66 ) | -0.48 ( -2.15 ) |
| Device malfunction | 340 | 3.29 ( 2.96 - 3.67 ) | 3.28 ( 534.23 ) | 3.26 ( 2.98 ) | 1.7 ( 0.04 ) |
| Injection site haemorrhage | 330 | 2.1 ( 1.88 - 2.34 ) | 2.09 ( 187.24 ) | 2.08 ( 1.9 ) | 1.06 ( -0.61 ) |
| Somnolence | 321 | 1.11 ( 0.99 - 1.24 ) | 1.11 ( 3.29 ) | 1.11 ( 1.01 ) | 0.15 ( -1.52 ) |
| Condition aggravated | 320 | 1.01 ( 0.9 - 1.12 ) | 1.01 ( 0.01 ) | 1.01 ( 0.92 ) | 0.01 ( -1.66 ) |
| Rash | 314 | 0.59 ( 0.53 - 0.66 ) | 0.59 ( 88.79 ) | 0.59 ( 0.54 ) | -0.75 ( -2.42 ) |
| Dyspnoea | 314 | 0.42 ( 0.38 - 0.47 ) | 0.42 ( 248.1 ) | 0.42 ( 0.39 ) | -1.24 ( -2.9 ) |
| Limb discomfort | 314 | 5.57 ( 4.98 - 6.23 ) | 5.55 ( 1149.07 ) | 5.46 ( 4.97 ) | 2.45 ( 0.78 ) |

Abbreviation: Asterisks (*) indicate statistically significant signals in algorithm; ROR, reporting odds ratio; PRR, proportional reporting ratio; EBGM, empirical Bayesian geometric mean; EBGM05, the lower limit of the 95% CI of EBGM; IC, information component; IC025, the lower limit of the 95% CI of the IC; CI, confidence interval; PT, preferred term.

**Supplementary Table 11**: Top 50 most frequent adverse events for Ofatumumab at the PT level in patients reported by medical personnels from FAERS data

| PT | Case numbers | ROR(95%CI) | PRR(χ^2^) | EBGM(EBGM05) | IC(IC025) |
| --- | --- | --- | --- | --- | --- |
| Pyrexia | 426 | 4.66 ( 4.23 - 5.13 ) | 4.54 ( 1181.23 ) | 4.53 ( 4.18 ) | 2.18 ( 0.51 ) |
| Fatigue | 312 | 2.14 ( 1.91 - 2.39 ) | 2.11 ( 183.92 ) | 2.11 ( 1.92 ) | 1.08 ( -0.59 ) |
| Headache | 276 | 2.4 ( 2.13 - 2.71 ) | 2.38 ( 221.37 ) | 2.37 ( 2.15 ) | 1.25 ( -0.42 ) |
| Chills | 236 | 9.22 ( 8.1 - 10.49 ) | 9.07 ( 1689.18 ) | 9.03 ( 8.1 ) | 3.17 ( 1.51 ) |
| Influenza like illness | 214 | 14.72 ( 12.86 - 16.86 ) | 14.5 ( 2670.78 ) | 14.39 ( 12.85 ) | 3.85 ( 2.18 ) |
| Pneumonia | 210 | 2.52 ( 2.2 - 2.89 ) | 2.5 ( 190.04 ) | 2.5 ( 2.23 ) | 1.32 ( -0.35 ) |
| Febrile neutropenia | 194 | 7.46 ( 6.47 - 8.6 ) | 7.36 ( 1064.59 ) | 7.34 ( 6.51 ) | 2.88 ( 1.21 ) |
| Drug ineffective | 193 | 0.81 ( 0.7 - 0.94 ) | 0.81 ( 8.27 ) | 0.81 ( 0.72 ) | -0.3 ( -1.96 ) |
| Pain | 183 | 1.9 ( 1.65 - 2.2 ) | 1.89 ( 77.39 ) | 1.89 ( 1.67 ) | 0.92 ( -0.75 ) |
| Multiple sclerosis relapse | 169 | 14 ( 12.02 - 16.3 ) | 13.83 ( 1997.39 ) | 13.73 ( 12.09 ) | 3.78 ( 2.11 ) |
| Nausea | 166 | 1.03 ( 0.89 - 1.2 ) | 1.03 ( 0.17 ) | 1.03 ( 0.91 ) | 0.05 ( -1.62 ) |
| Covid-19 | 150 | 3.96 ( 3.37 - 4.65 ) | 3.93 ( 327.63 ) | 3.92 ( 3.43 ) | 1.97 ( 0.31 ) |
| Vomiting | 124 | 1.2 ( 1 - 1.43 ) | 1.19 ( 3.95 ) | 1.19 ( 1.03 ) | 0.26 ( -1.41 ) |
| Accidental exposure to product | 123 | 6.4 ( 5.35 - 7.64 ) | 6.35 ( 552.86 ) | 6.33 ( 5.45 ) | 2.66 ( 1 ) |
| Malaise | 117 | 1.43 ( 1.2 - 1.72 ) | 1.43 ( 15.2 ) | 1.43 ( 1.23 ) | 0.52 ( -1.15 ) |
| Asthenia | 112 | 1.52 ( 1.26 - 1.82 ) | 1.51 ( 19.43 ) | 1.51 ( 1.29 ) | 0.59 ( -1.07 ) |
| Death | 111 | 0.55 ( 0.46 - 0.66 ) | 0.55 ( 40.59 ) | 0.55 ( 0.47 ) | -0.85 ( -2.52 ) |
| Rash | 111 | 1.09 ( 0.91 - 1.32 ) | 1.09 ( 0.84 ) | 1.09 ( 0.93 ) | 0.12 ( -1.54 ) |
| Dizziness | 107 | 1.22 ( 1.01 - 1.48 ) | 1.22 ( 4.29 ) | 1.22 ( 1.04 ) | 0.29 ( -1.38 ) |
| Urinary tract infection | 105 | 2.74 ( 2.26 - 3.32 ) | 2.72 ( 114.77 ) | 2.72 ( 2.32 ) | 1.44 ( -0.22 ) |
| Dyspnoea | 105 | 0.84 ( 0.69 - 1.01 ) | 0.84 ( 3.31 ) | 0.84 ( 0.71 ) | -0.25 ( -1.92 ) |
| Neutropenia | 94 | 1.74 ( 1.42 - 2.14 ) | 1.74 ( 29.54 ) | 1.74 ( 1.47 ) | 0.8 ( -0.87 ) |
| Inappropriate schedule of product administration | 94 | 3.37 ( 2.75 - 4.13 ) | 3.36 ( 155.61 ) | 3.35 ( 2.83 ) | 1.75 ( 0.08 ) |
| Infection | 85 | 2.29 ( 1.85 - 2.83 ) | 2.28 ( 60.96 ) | 2.28 ( 1.9 ) | 1.19 ( -0.48 ) |
| Gait disturbance | 85 | 2.71 ( 2.19 - 3.36 ) | 2.7 ( 91.23 ) | 2.7 ( 2.26 ) | 1.43 ( -0.23 ) |
| Hypoaesthesia | 84 | 3.05 ( 2.46 - 3.79 ) | 3.04 ( 115.1 ) | 3.04 ( 2.54 ) | 1.6 ( -0.06 ) |
| Diarrhoea | 80 | 0.55 ( 0.44 - 0.69 ) | 0.55 ( 29.19 ) | 0.55 ( 0.46 ) | -0.85 ( -2.52 ) |
| Arthralgia | 79 | 0.9 ( 0.72 - 1.13 ) | 0.9 ( 0.83 ) | 0.9 ( 0.75 ) | -0.15 ( -1.81 ) |
| Maternal exposure during pregnancy | 79 | 2.99 ( 2.39 - 3.73 ) | 2.97 ( 103.59 ) | 2.97 ( 2.47 ) | 1.57 ( -0.1 ) |
| Condition aggravated | 76 | 1.03 ( 0.82 - 1.29 ) | 1.03 ( 0.07 ) | 1.03 ( 0.85 ) | 0.04 ( -1.62 ) |
| Myalgia | 76 | 2.2 ( 1.76 - 2.76 ) | 2.19 ( 49.44 ) | 2.19 ( 1.82 ) | 1.13 ( -0.53 ) |
| Cough | 74 | 1.35 ( 1.07 - 1.7 ) | 1.35 ( 6.64 ) | 1.35 ( 1.11 ) | 0.43 ( -1.24 ) |
| Device malfunction | 73 | 6.12 ( 4.86 - 7.71 ) | 6.09 ( 309.86 ) | 6.07 ( 5.01 ) | 2.6 ( 0.94 ) |
| Pain in extremity | 71 | 1.28 ( 1.02 - 1.62 ) | 1.28 ( 4.37 ) | 1.28 ( 1.05 ) | 0.36 ( -1.31 ) |
| Urticaria | 70 | 1.84 ( 1.46 - 2.33 ) | 1.84 ( 26.7 ) | 1.84 ( 1.51 ) | 0.88 ( -0.79 ) |
| Back pain | 68 | 1.51 ( 1.19 - 1.92 ) | 1.51 ( 11.72 ) | 1.51 ( 1.24 ) | 0.59 ( -1.07 ) |
| Lymphocyte count decreased | 68 | 11.25 ( 8.85 - 14.28 ) | 11.19 ( 627.36 ) | 11.13 ( 9.11 ) | 3.48 ( 1.81 ) |
| Pruritus | 66 | 0.88 ( 0.69 - 1.12 ) | 0.88 ( 1.14 ) | 0.88 ( 0.72 ) | -0.19 ( -1.86 ) |
| Feeling abnormal | 64 | 2.07 ( 1.62 - 2.65 ) | 2.07 ( 35.37 ) | 2.07 ( 1.68 ) | 1.05 ( -0.62 ) |
| Product dose omission issue | 64 | 1.64 ( 1.28 - 2.1 ) | 1.64 ( 15.97 ) | 1.64 ( 1.33 ) | 0.71 ( -0.95 ) |
| Muscular weakness | 63 | 2.8 ( 2.19 - 3.59 ) | 2.8 ( 72.63 ) | 2.79 ( 2.27 ) | 1.48 ( -0.18 ) |
| Sepsis | 62 | 1.74 ( 1.36 - 2.24 ) | 1.74 ( 19.59 ) | 1.74 ( 1.41 ) | 0.8 ( -0.87 ) |
| Infusion related reaction | 59 | 2.64 ( 2.05 - 3.41 ) | 2.64 ( 59.88 ) | 2.63 ( 2.13 ) | 1.4 ( -0.27 ) |
| Fall | 56 | 0.83 ( 0.64 - 1.07 ) | 0.83 ( 2.05 ) | 0.83 ( 0.66 ) | -0.27 ( -1.94 ) |
| Hypersensitivity | 54 | 1.42 ( 1.09 - 1.86 ) | 1.42 ( 6.75 ) | 1.42 ( 1.14 ) | 0.51 ( -1.16 ) |
| Muscle spasms | 54 | 1.67 ( 1.28 - 2.19 ) | 1.67 ( 14.59 ) | 1.67 ( 1.34 ) | 0.74 ( -0.93 ) |
| Lower respiratory tract infection | 53 | 4.9 ( 3.74 - 6.42 ) | 4.89 ( 163.49 ) | 4.88 ( 3.89 ) | 2.29 ( 0.62 ) |
| Paraesthesia | 53 | 1.62 ( 1.24 - 2.12 ) | 1.62 ( 12.48 ) | 1.62 ( 1.29 ) | 0.69 ( -0.97 ) |
| Tremor | 50 | 1.69 ( 1.28 - 2.24 ) | 1.69 ( 14.17 ) | 1.69 ( 1.34 ) | 0.76 ( -0.91 ) |
| Neutropenic sepsis | 49 | 16.91 ( 12.75 - 22.41 ) | 16.85 ( 723.44 ) | 16.69 ( 13.18 ) | 4.06 ( 2.39 ) |

Abbreviation: Asterisks (*) indicate statistically significant signals in algorithm; ROR, reporting odds ratio; PRR, proportional reporting ratio; EBGM, empirical Bayesian geometric mean; EBGM05, the lower limit of the 95% CI of EBGM; IC, information component; IC025, the lower limit of the 95% CI of the IC; CI, confidence interval; PT, preferred term.

**Supplementary Table 12**: Top 50 most frequent adverse events for Ofatumumab excluding common medication co-usage at the PT level from FAERS data

| PT | Case numbers | ROR(95%CI) | PRR(χ^2^) | EBGM(EBGM05) | IC(IC025) |
| --- | --- | --- | --- | --- | --- |
| Fatigue | 4460 | 4.1 ( 3.98 - 4.23 ) | 3.94 ( 9848.21 ) | 3.92 ( 3.82 ) | 1.97 ( 0.3 ) |
| Headache | 3886 | 4.48 ( 4.34 - 4.63 ) | 4.32 ( 9954.77 ) | 4.3 ( 4.18 ) | 2.1 ( 0.44 ) |
| Chills | 3050 | 19.71 ( 19 - 20.45 ) | 19.05 ( 50524.73 ) | 18.45 ( 17.89 ) | 4.21 ( 2.54 ) |
| Pain | 2897 | 3.18 ( 3.07 - 3.3 ) | 3.11 ( 4167.25 ) | 3.1 ( 3 ) | 1.63 ( -0.03 ) |
| Pyrexia | 2857 | 6.12 ( 5.9 - 6.35 ) | 5.95 ( 11708.4 ) | 5.9 ( 5.72 ) | 2.56 ( 0.89 ) |
| Influenza like illness | 2360 | 20.68 ( 19.84 - 21.56 ) | 20.14 ( 41484.61 ) | 19.47 ( 18.8 ) | 4.28 ( 2.62 ) |
| Nausea | 1421 | 1.3 ( 1.23 - 1.37 ) | 1.29 ( 94.02 ) | 1.29 ( 1.23 ) | 0.37 ( -1.3 ) |
| Covid-19 | 1201 | 4.47 ( 4.22 - 4.73 ) | 4.42 ( 3159.81 ) | 4.39 ( 4.19 ) | 2.13 ( 0.47 ) |
| Asthenia | 1119 | 2.14 ( 2.02 - 2.27 ) | 2.12 ( 666.17 ) | 2.12 ( 2.02 ) | 1.08 ( -0.58 ) |
| Hypoaesthesia | 1027 | 4.85 ( 4.56 - 5.16 ) | 4.81 ( 3078.19 ) | 4.77 ( 4.53 ) | 2.26 ( 0.59 ) |
| Gait disturbance | 1021 | 3.57 ( 3.35 - 3.79 ) | 3.54 ( 1851.58 ) | 3.52 ( 3.34 ) | 1.82 ( 0.15 ) |
| Multiple sclerosis relapse | 1018 | 9.38 ( 8.82 - 9.99 ) | 9.28 ( 7410.1 ) | 9.15 ( 8.68 ) | 3.19 ( 1.53 ) |
| Feeling abnormal | 1013 | 2.89 ( 2.72 - 3.07 ) | 2.87 ( 1230.12 ) | 2.86 ( 2.71 ) | 1.51 ( -0.15 ) |
| Malaise | 961 | 1.46 ( 1.37 - 1.55 ) | 1.45 ( 134.86 ) | 1.45 ( 1.37 ) | 0.53 ( -1.13 ) |
| Dizziness | 960 | 1.39 ( 1.3 - 1.48 ) | 1.38 ( 102.58 ) | 1.38 ( 1.31 ) | 0.47 ( -1.2 ) |
| Drug ineffective | 870 | 0.44 ( 0.41 - 0.47 ) | 0.45 ( 612.7 ) | 0.45 ( 0.42 ) | -1.16 ( -2.83 ) |
| Pain in extremity | 866 | 1.99 ( 1.86 - 2.13 ) | 1.98 ( 419.39 ) | 1.97 ( 1.87 ) | 0.98 ( -0.68 ) |
| Nasopharyngitis | 858 | 3.19 ( 2.98 - 3.41 ) | 3.17 ( 1270.63 ) | 3.16 ( 2.98 ) | 1.66 ( -0.01 ) |
| Injection site pain | 834 | 2.01 ( 1.88 - 2.15 ) | 2 ( 416.27 ) | 1.99 ( 1.88 ) | 1 ( -0.67 ) |
| Arthralgia | 724 | 1.21 ( 1.13 - 1.31 ) | 1.21 ( 26.84 ) | 1.21 ( 1.14 ) | 0.28 ( -1.39 ) |
| Inappropriate schedule of product administration | 722 | 3.12 ( 2.9 - 3.36 ) | 3.1 ( 1027.37 ) | 3.09 ( 2.91 ) | 1.63 ( -0.04 ) |
| Myalgia | 713 | 3.06 ( 2.84 - 3.3 ) | 3.04 ( 975.64 ) | 3.03 ( 2.85 ) | 1.6 ( -0.07 ) |
| Accidental exposure to product | 693 | 5.34 ( 4.95 - 5.76 ) | 5.31 ( 2402.63 ) | 5.27 ( 4.94 ) | 2.4 ( 0.73 ) |
| Back pain | 693 | 2.07 ( 1.92 - 2.23 ) | 2.06 ( 377.78 ) | 2.06 ( 1.93 ) | 1.04 ( -0.63 ) |
| Cough | 665 | 1.66 ( 1.54 - 1.79 ) | 1.66 ( 173.38 ) | 1.65 ( 1.55 ) | 0.73 ( -0.94 ) |
| Fall | 659 | 1.38 ( 1.28 - 1.49 ) | 1.38 ( 69.06 ) | 1.38 ( 1.29 ) | 0.46 ( -1.2 ) |
| Vomiting | 658 | 1.03 ( 0.95 - 1.11 ) | 1.03 ( 0.44 ) | 1.03 ( 0.96 ) | 0.04 ( -1.63 ) |
| Muscle spasms | 611 | 2.3 ( 2.12 - 2.49 ) | 2.29 ( 443.54 ) | 2.28 ( 2.14 ) | 1.19 ( -0.47 ) |
| Muscular weakness | 610 | 3.77 ( 3.48 - 4.08 ) | 3.75 ( 1223.5 ) | 3.73 ( 3.49 ) | 1.9 ( 0.23 ) |
| Product dose omission issue | 608 | 1.82 ( 1.68 - 1.97 ) | 1.81 ( 221.17 ) | 1.81 ( 1.69 ) | 0.86 ( -0.81 ) |
| Urinary tract infection | 601 | 2.46 ( 2.27 - 2.66 ) | 2.45 ( 512.52 ) | 2.44 ( 2.28 ) | 1.29 ( -0.38 ) |
| Paraesthesia | 597 | 2.67 ( 2.46 - 2.89 ) | 2.66 ( 615.79 ) | 2.65 ( 2.48 ) | 1.41 ( -0.26 ) |
| Tremor | 577 | 2.52 ( 2.32 - 2.73 ) | 2.51 ( 522.2 ) | 2.5 ( 2.34 ) | 1.32 ( -0.34 ) |
| Illness | 544 | 4.58 ( 4.2 - 4.98 ) | 4.55 ( 1497.79 ) | 4.52 ( 4.21 ) | 2.18 ( 0.51 ) |
| Balance disorder | 542 | 4.3 ( 3.95 - 4.68 ) | 4.28 ( 1352.31 ) | 4.25 ( 3.96 ) | 2.09 ( 0.42 ) |
| Memory impairment | 494 | 2.41 ( 2.2 - 2.63 ) | 2.4 ( 403.09 ) | 2.4 ( 2.22 ) | 1.26 ( -0.41 ) |
| Oropharyngeal pain | 480 | 3.43 ( 3.13 - 3.75 ) | 3.42 ( 816.15 ) | 3.4 ( 3.15 ) | 1.77 ( 0.1 ) |
| Injection site bruising | 448 | 4.07 ( 3.71 - 4.47 ) | 4.06 ( 1025.9 ) | 4.04 ( 3.73 ) | 2.01 ( 0.35 ) |
| Migraine | 423 | 3.17 ( 2.88 - 3.49 ) | 3.16 ( 623.17 ) | 3.15 ( 2.91 ) | 1.66 ( -0.01 ) |
| Insomnia | 421 | 1.11 ( 1.01 - 1.23 ) | 1.11 ( 4.91 ) | 1.11 ( 1.03 ) | 0.16 ( -1.51 ) |
| Diarrhoea | 417 | 0.45 ( 0.41 - 0.5 ) | 0.45 ( 277.64 ) | 0.45 ( 0.42 ) | -1.14 ( -2.81 ) |
| Device malfunction | 404 | 4.3 ( 3.89 - 4.74 ) | 4.28 ( 1008.89 ) | 4.25 ( 3.92 ) | 2.09 ( 0.42 ) |
| Pruritus | 402 | 0.8 ( 0.72 - 0.88 ) | 0.8 ( 20.93 ) | 0.8 ( 0.73 ) | -0.33 ( -1.99 ) |
| Dyspnoea | 398 | 0.49 ( 0.45 - 0.54 ) | 0.5 ( 206.67 ) | 0.5 ( 0.46 ) | -1.01 ( -2.68 ) |
| Rash | 394 | 0.65 ( 0.59 - 0.72 ) | 0.65 ( 74.44 ) | 0.65 ( 0.6 ) | -0.62 ( -2.29 ) |
| Condition aggravated | 382 | 0.94 ( 0.85 - 1.04 ) | 0.94 ( 1.51 ) | 0.94 ( 0.86 ) | -0.09 ( -1.76 ) |
| Rhinorrhoea | 372 | 4.02 ( 3.63 - 4.45 ) | 4 ( 832.74 ) | 3.98 ( 3.65 ) | 1.99 ( 0.33 ) |
| Pneumonia | 357 | 0.78 ( 0.7 - 0.86 ) | 0.78 ( 23.01 ) | 0.78 ( 0.71 ) | -0.36 ( -2.03 ) |
| Injection site haemorrhage | 354 | 3.23 ( 2.91 - 3.58 ) | 3.22 ( 538.78 ) | 3.21 ( 2.94 ) | 1.68 ( 0.01 ) |
| Influenza | 339 | 2.14 ( 1.93 - 2.39 ) | 2.14 ( 205.46 ) | 2.14 ( 1.95 ) | 1.09 ( -0.57 ) |

Abbreviation: Asterisks (*) indicate statistically significant signals in algorithm; ROR, reporting odds ratio; PRR, proportional reporting ratio; EBGM, empirical Bayesian geometric mean; EBGM05, the lower limit of the 95% CI of EBGM; IC, information component; IC025, the lower limit of the 95% CI of the IC; CI, confidence interval; PT, preferred term.
